# Supplementary material for: A systems‐level study reveals host‐targeted repurposable drugs against SARS‐CoV‐2 infection
Source: Mol Syst Biol. 2021 Aug 2;17(8):e10239. doi: 10.15252/msb.202110239 (PMC8328275; doi:10.15252/msb.202110239)
Supplement: Supplementary file 1 — Appendix [file MSB-17-e10239-s002.pdf]

APPENDIX

for

A systems-level study reveals host-targeted repurposable drugs against SARS-CoV-2 infection

Systems-level analysis reveals anti-SARS-CoV-2 repurposable drugs and compounds targeting the host cell

Fangyuan Chen[†,1,2], Qingya Shi[†,1,2], Fen Pei[†,1,3], Andreas Vogt[†,1,3], Rebecca A. Porritt[4,8] Gustavo Garcia Jr[5], Angela C. Gomez[4], Mary H. Cheng[1], Mark Schurdak[1,3], Bing Liu[1], Stephen Y. Chan[6,7], Vaithilingaraja Arumugaswami[5], Andrew M Stern[1,3], D Lansing Taylor[1,3], Moshe Arditi[4,8], and Ivet Bahar[*,1,3]

[1]Department of Computational and Systems Biology, School of Medicine, University of Pittsburgh, Pittsburgh, PA, 15213, USA; [2]School of Medicine, Tsinghua University, Beijing, 100084, China; [3]University of Pittsburgh Drug Discovery Institute, Pittsburgh, PA 15213; [4]Department of Pediatrics, Division of Pediatric Infectious Diseases and Immunology, Cedars-Sinai Medical Center, Los Angeles, CA, 90048; [5]Department of Molecular and Medical Pharmacology, David Geffen School of Medicine, University of California and Eli and Edythe Broad Center of Regenerative Medicine and Stem Cell Research, University of California, Los Angeles; [6]Pittsburgh Heart, Lung, Blood, and Vascular Medicine Institute, and [7]Division of Cardiology, Department of Medicine, University of Pittsburgh Medical Center, Pittsburgh, PA, 15217; [8]Biomedical Sciences, Infectious and Immunologic Diseases Research Center, Cedars-Sinai Medical Center, Los Angeles, CA, 90048

[†] These authors made equal contribution

[*] **Correspondence:** Dr. Ivet Bahar, bahar@pitt.edu

TABLE OF CONTENTS

Section	Pages
**Supplementary Figures**	**3-8**
Figure S1	3
Figure S2	4-5
Figure S3	6
Figure S4	7
Figure S5	8
Figure S6	9
Figure S7	10
**Supplementary Tables**	**10-36~~2~~**
Table S1	10-13
Table S2	14-18
Table S3	19-27
Table S4	28-29~~28-32~~
Table S5	30-31
Table S6	32-36

SUPPLEMENTARY FIGURES

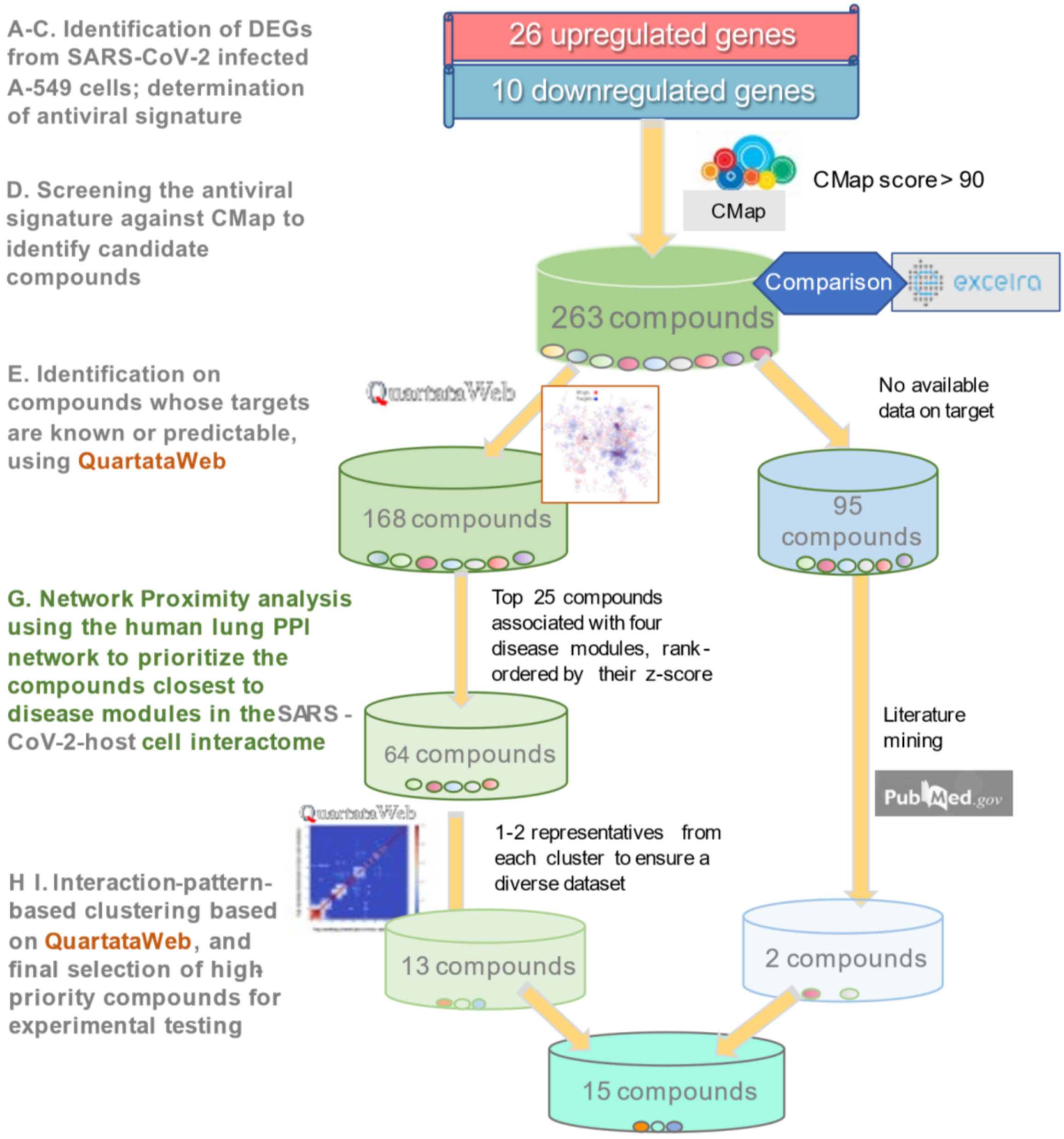

Figure S1. Identification of candidate compounds/drugs, their prioritization and final selection of a small set for experimental tests, illustrated for Dataset 1 (related to Figure 1). The flow diagram depicts the number of compounds/drugs extracted at various stages, indicated by the Steps A-I (*on the left*) consistent with **Figure 1** panels **A-I**. The original analysis of transcriptomics data from A549 cells leads to 36 DEGs, whose antiviral signature screened against CMap database identifies 263 candidate compounds. Comparison with Excelra DB shows those (10 of them) already listed therein. Of these 263 compounds, 168 have target information available and/or predictable in QuartataWeb (Li et al, 2020) – an interface that utilizes as input DrugBank and STITCH database. Two different paths are then followed, for the respective subsets of 168 and 95 compounds. In the former case, the targets of these 168 compounds are subjected to network proximity analysis with respect to four disease modules in SARS-CoV-2-host interactome, using

BioSNAP human lung PPI network; this analysis yields 64 compounds, which, upon clustering (using QuartataWeb) to select representatives, are reduced to 13 high-priority compounds. The latter set of 95 compounds are manually analyzed to select two compounds, leading to a total set of 15 high priority compounds that have been further investigated in experiments. The diagram depicts the protocol for antiviral compounds. In the case of anti-cytokine compounds, the same schema without step G is adopted.

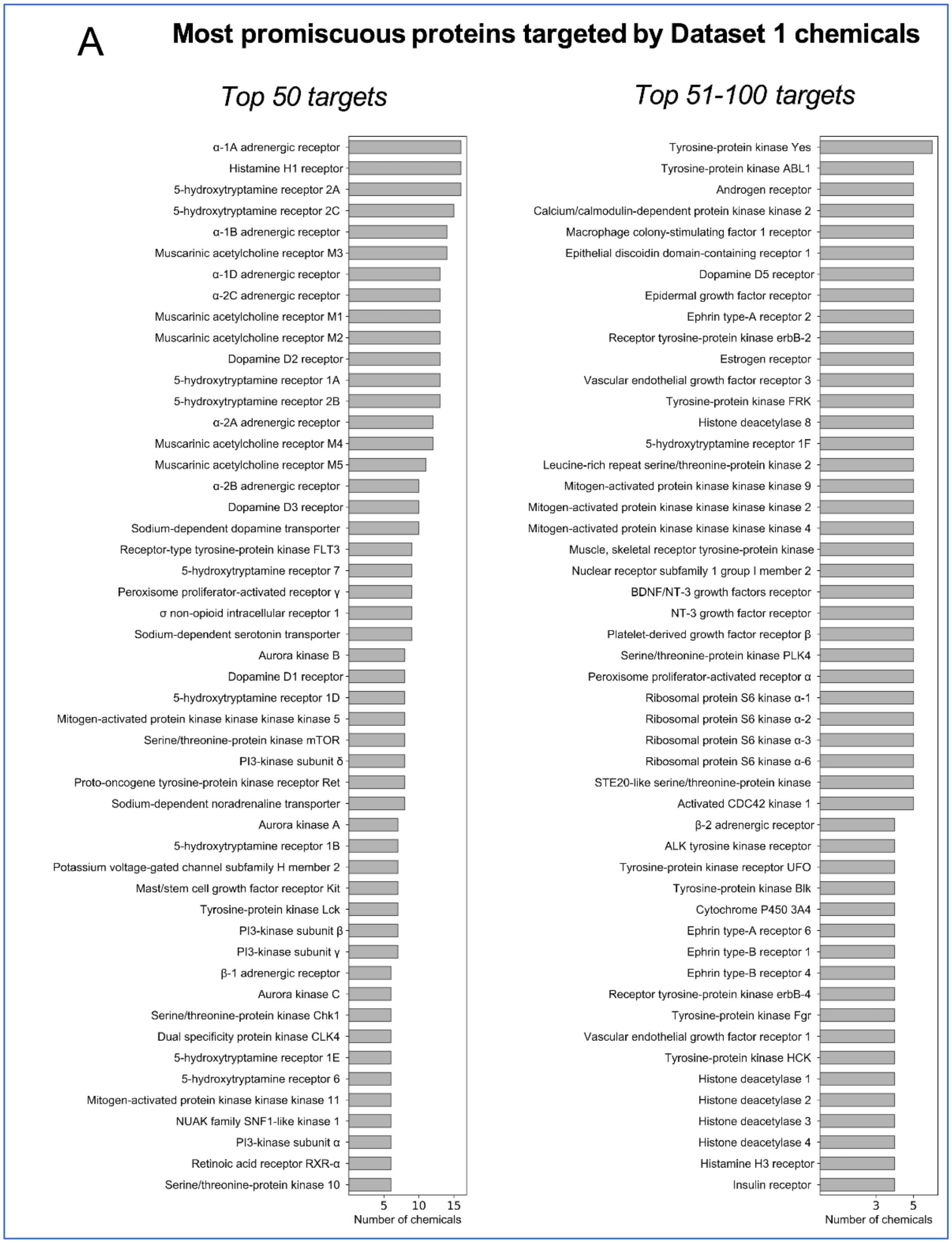

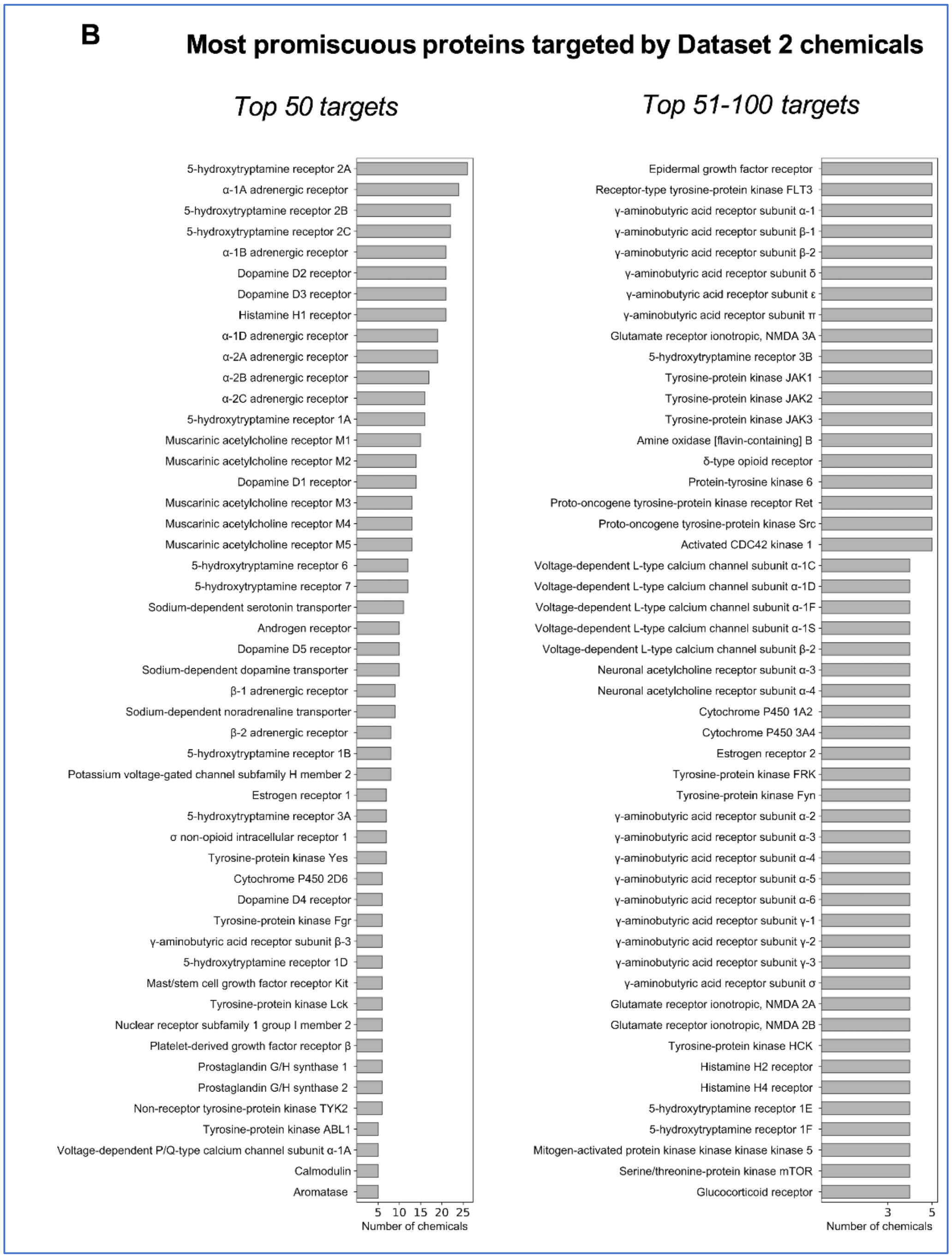

Figure S2. Host cell proteins targeted by potential antiviral (Dataset 1) or anti-cytokine (Dataset 2) compounds/drugs, rank-ordered by their promiscuity, related to Figures 1E and 4. Promiscuity refers to the number of predicted compounds/drugs (also called chemicals) that target the protein. Panel A lists the top 100 targets corresponding to Dataset 1 compounds/drugs. The ordinate lists the proteins, and the horizontal bars (abscissa) show the corresponding number of compounds. Panel **B** is same as Panel **A**, for compounds deduced from Dataset 2.

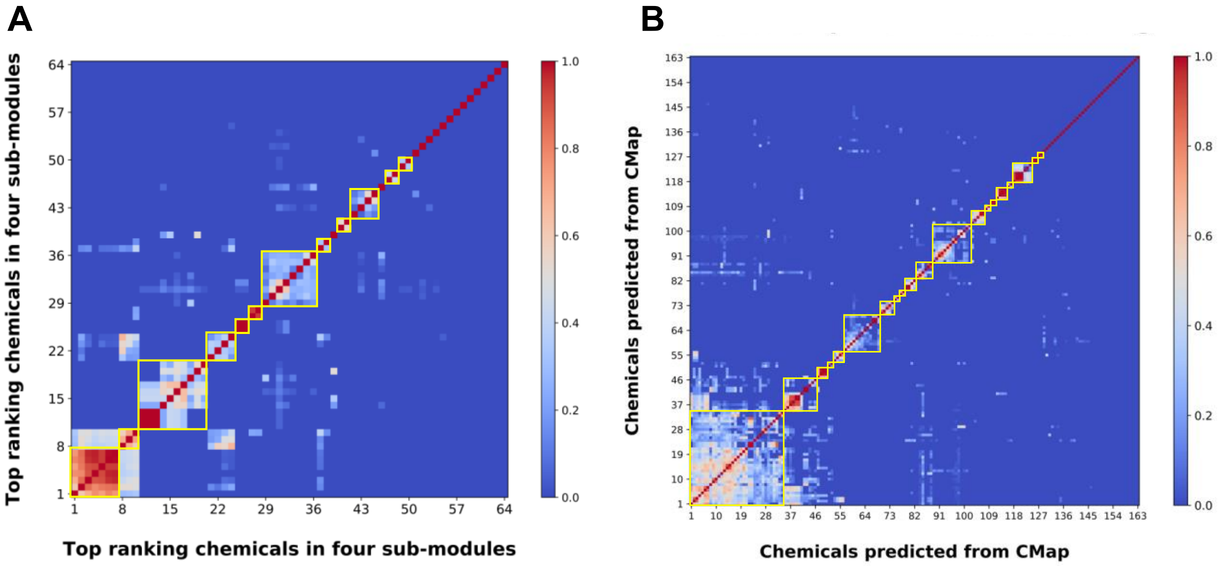

Figure S3. Interaction-pattern-based clustering of top-ranking compounds from four modules and chemicals targeting immune response, related to Figure 1G and H, and Tables 3 and 4. (A) Results for 64 compounds (or chemicals) identified to yield closest proximity to four selected modules. The compounds are clustered based on their interaction patterns with their targets listed in QuartataWeb. 12 main clusters (clusters 1- 8, 10, 11, 13 and 14, from *left* to *right*, delimited by *yellow* squares) contain two or more compounds each; 16 chemicals do not belong to any cluster. From each of cluster, we selected up to two chemicals based on their side effects and MOA. (B) Clustering of 163 chemicals proposed to modulate the immune response, based on anti-cytokine signature gene derived from infected A546-Ace2 cells. The chemicals are clustered based on their interaction patterns reported in DrugBank or STITCH. We distinguish 20 main clusters (marked by *yellow* squares) which contain two or more chemicals, and 35 additional chemicals that do not form clusters.

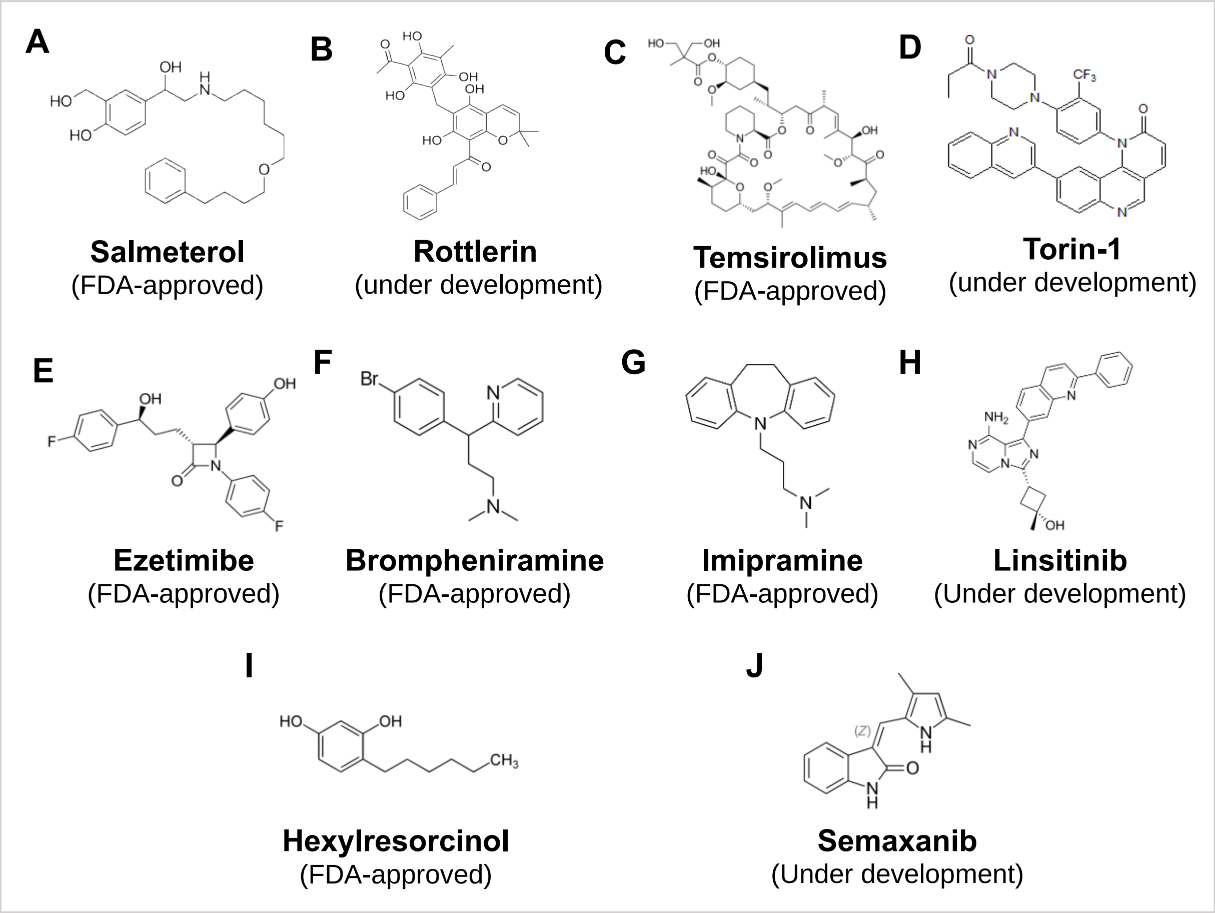

Figure S4. Structure of ten chemicals tested for SARS-CoV-2 infection inhibitory activity *in vitro*, related to Table 3 and Figure 4. Structures of salmeterol, rottlerin, temsirolimus, torin-1, ezetimibe, brompheniramine, imipramine, linsitinib, hexylresorcinol, and semaxanib, selected for *in vitro* assays.

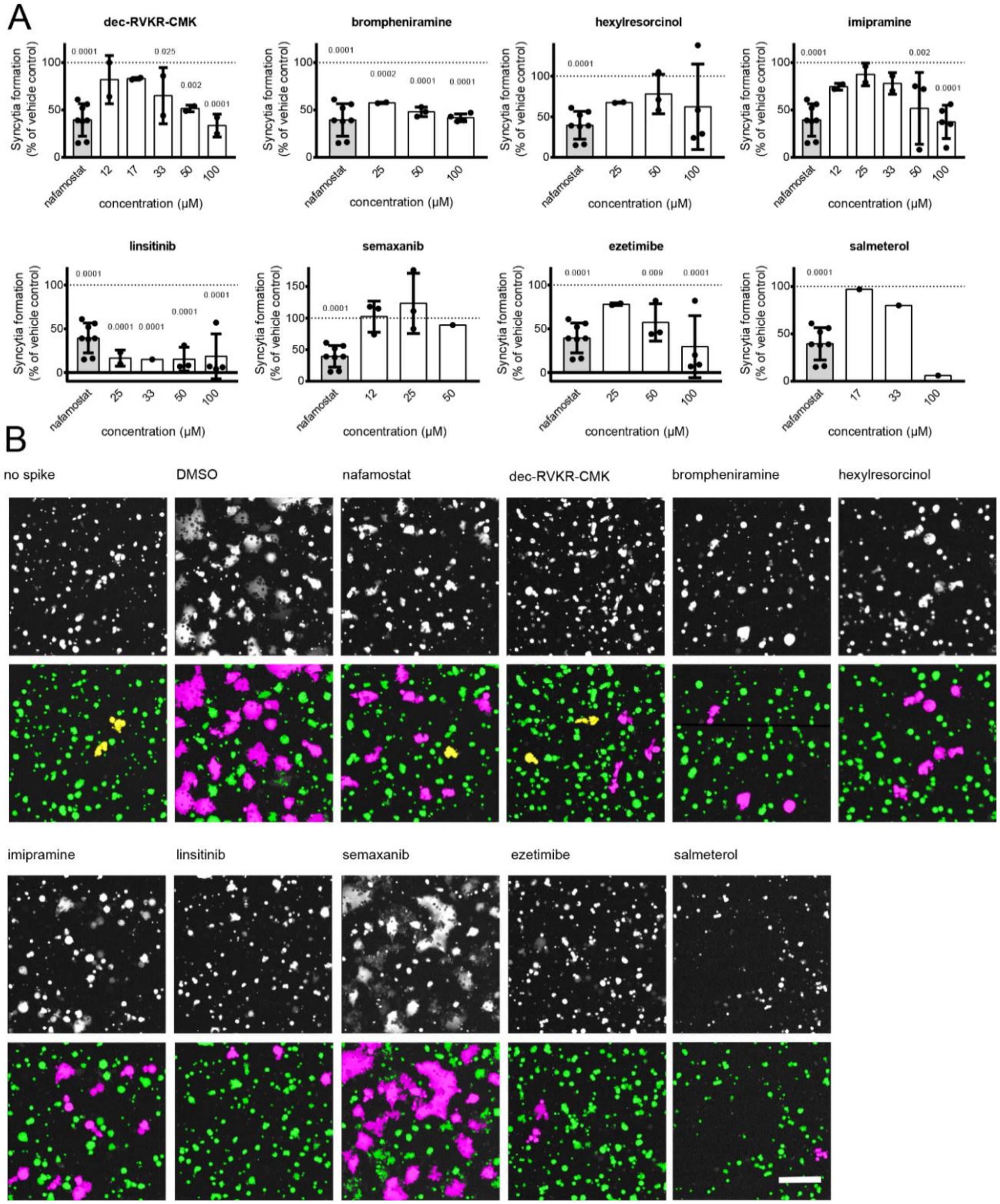

Figure S5. Results from syncytia experiments in HEK293 cells, related to Figure 6. HEK293 acceptor cells transfected with or without ACE2 and TMPRSS2 were seeded in 384 well plates, pretreated with 7-point gradients of test compounds for 1-2 h, and co-cultured for 4 hours with HEK293 donor cells expressing SARS-CoV-2 spike and GFP, or donor cells expressing GFP only (*no spike*). Images of GFP-positive objects were acquired on a confocal high-content imager and analyzed for syncytia formation and total GFP as a measure of cytotoxicity, using a CNT algorithm as described in the Methods Section. A. Quantification of syncytia formation. B. Representative images illustrating syncytia phenotype and compound activity. Images are shown at the 100 μM condition except nafamostat (5.5 μM), semaxanib

(50 µM), and linsitinib (25 µM). *Upper panels*, raw fluorescence micrographs; *lower panels*, images with CNT overlay. GFP positive objects that met the criteria for syncytia are colored *purple*; cellular aggregates that are not syncytia are shown in *yellow*. Scale bar, 100 µm. *No spike*, donor cells expressing GFP only. Numbers indicate p-values obtained by one-way ANOVA (non-matched, unpaired) with Dunnett's multiple comparisons test in Graph Pad Prism (v7.00) compared with vehicle control (*dotted line*). No p-value, $p > 0.05$. *Bars* and *errors* represent the means ± SD from multiple independent biological repeats, each performed in quadruplicate. No error bars for $n = 1$.

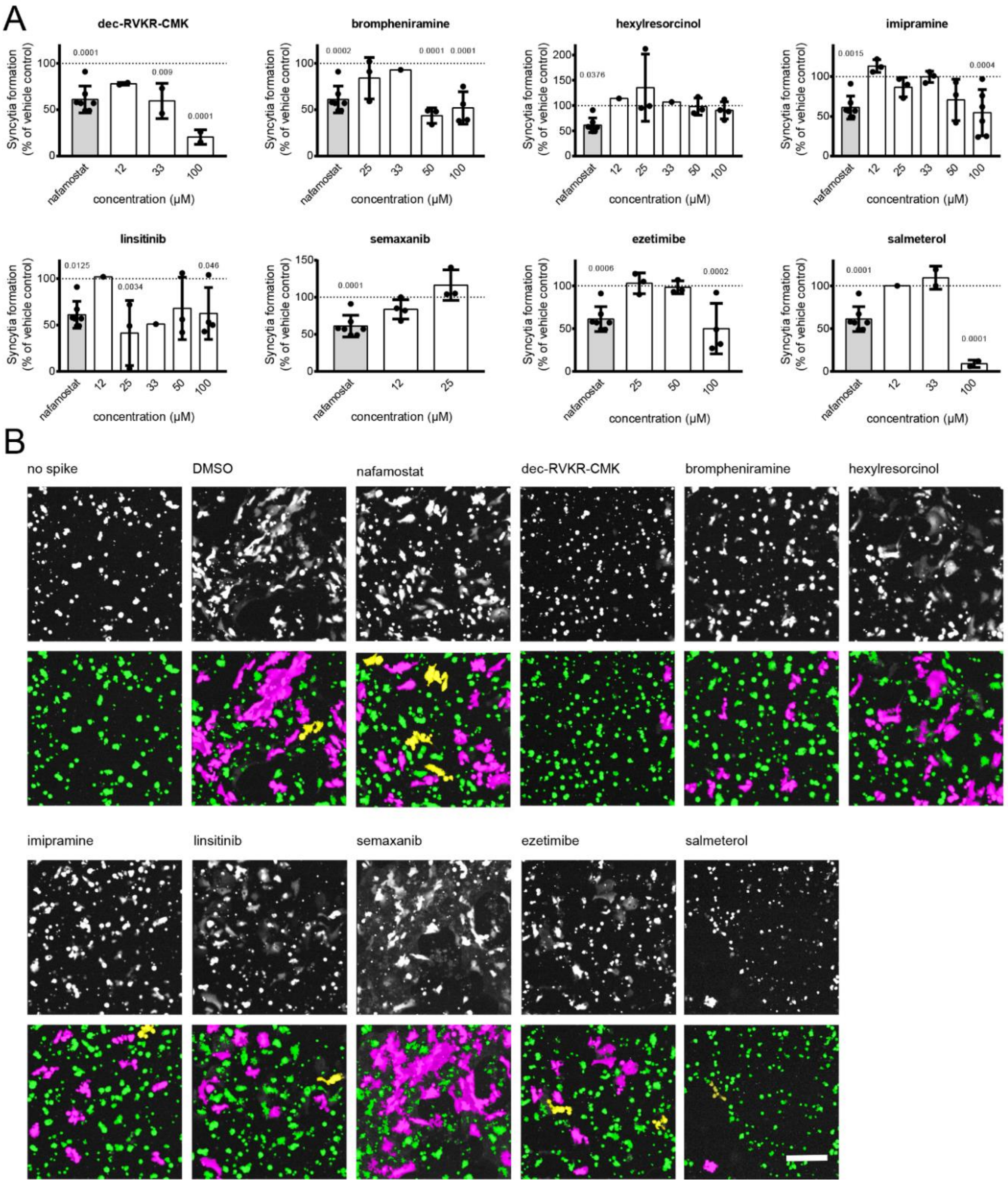

Figure S6. **Results from syncytia experiments in Calu-3 cells, related to Figure 6.** Calu-3 acceptor cells were seeded in 384 well plates, pretreated with compounds for 1-2 h, and co-cultured for 4 hours with HEK293 donor cells expressing SARS-CoV-2 spike and GFP. Images of GFP-positive objects were acquired on a confocal high-content imager and analyzed for syncytia formation using a CNT algorithm as described in the Methods. **A**. Quantification of syncytia formation. **B**. Representative images illustrating syncytia phenotype and compound activity. Images are shown at the 100 µM condition except nafamostat (5.5 µM), semaxanib (50 µM), and linsitinib (25 µM). *Upper panels*, raw fluorescence micrographs; *lower panels*, images with CNT overlay. GFP positive objects that met the criteria for syncytia are colored *purple*; cellular aggregates that are not syncytia are shown in *yellow*. Scale bar, 100 µm. *No spike*, donor cells expressing GFP only. Numbers indicate p-values obtained by one-way ANOVA (non-matched, unpaired) with Dunnett's multiple comparisons test in Graph Pad Prism (v7.00) compared with vehicle control (*dotted line*). No p-value, $p > 0.05$. *Bars* and *errors* represent the means ± SD from multiple independent biological repeats, each performed in quadruplicate. No error bars, n = 1.

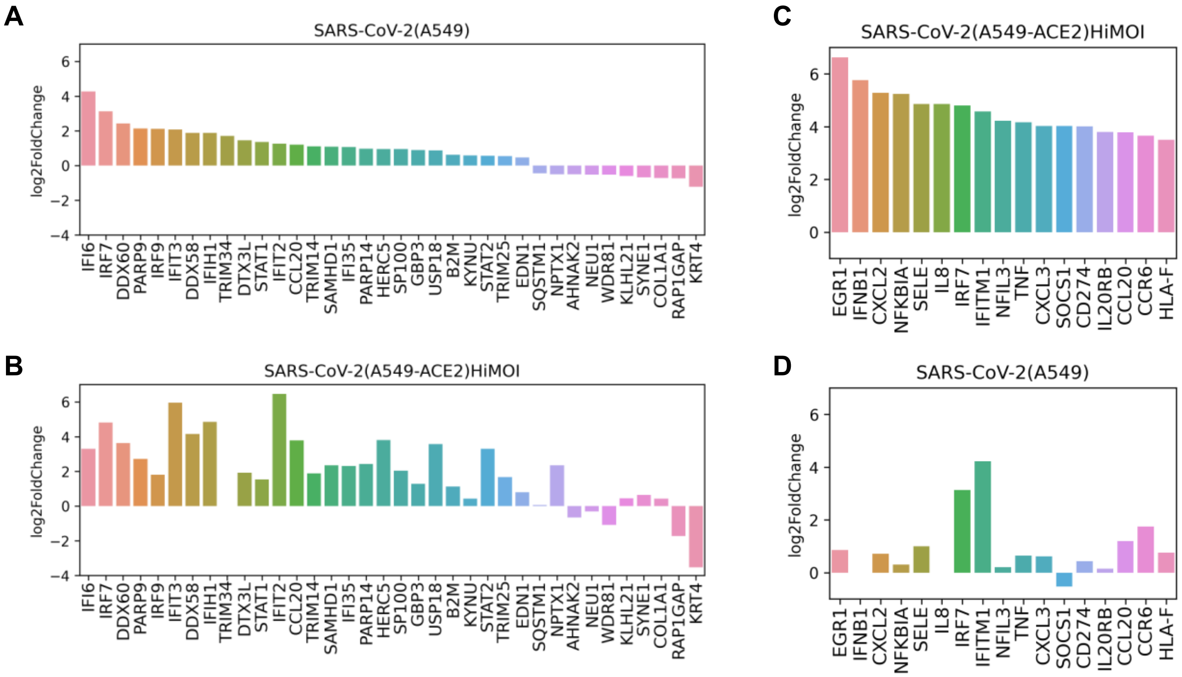

Figure S7. **Comparison of the behavior of A546 and A546-ACE2 cells vis-à-vis the expression levels of the genes that have been adopted for defining antiviral and anticytokine signatures**. **A**. The 36 genes of antiviral signature expression in A549 cells (same as **Figure 2C**). **B**. The 36 genes of antiviral signature expression in A549-ACE2 cells. **C**. The 17 genes of anti-inflammatory signature expression in A549-ACE2 cells (same as **Figure 2D**). **D**. The 17 genes of anti-inflammatory signature expression in A549 cells.

SUPPLEMENTARY TABLES

Table S1. 120 differentially expressed genes (DEGs) in SARS-CoV-2-infected A549 cells

Gene name	Protein name	log_2 fold change	$P_{adjusted}$
**Upregulated 100 genes in SARS-CoV-2-infected A549 cells**			
MX1	IFN-induced GTP-binding protein Mx1	5.20	1.16E-93
IFI44	IFN-induced protein 44 (Fragment)	4.59	6.80E-04
IFIT1	IFN-induced protein with tetratricopeptide repeats 1 (IFIT-1) (IFN-induced 56 kDa protein) (IFI-56K) (P56)	4.43	6.18E-96
IFI6	IFN α-inducible protein 6	4.27	1.01E-145
OAS2	2'-5'-oligoadenylate synthase 2	4.25	1.49E-06
IFITM1	IFN induced transmembrane protein 1 (9-27), isoform CRA_a	4.22	3.12E-05
ISG15	Ubiquitin-like protein ISG15 (Fragment)	3.80	6.21E-85
IFI27	IFNα-inducible protein 27, mitochondrial (Fragment)	3.54	8.09E-14
IRF7	IFN regulatory factor 7, isoform CRA_a	3.13	5.69E-43
PTPRE	Receptor-type tyrosine-protein phosphatase epsilon (Protein-tyrosine phosphatase epsilon) (R-PTP-epsilon) (EC 3.1.3.48)	3.02	3.36E-10
OASL	2'-5'-oligoadenylate synthase-like protein (Fragment)	2.53	8.17E-07
DDX60	ATP-dependent RNA helicase DDX60	2.42	1.43E-25
CMPK2	Mitochondrial cytidine monophosphate (UMP-CMP) kinase 2 (Fragment)	2.23	7.66E-03
PARP9	Protein mono-ADP-ribosyltransferase PARP9 (Fragment)	2.13	4.32E-43
IRF9	IFN regulatory factor 9	2.11	6.73E-42
IFIT3	IFN-induced protein with tetratricopeptide repeats 3 (IFIT-3) (CIG49) (ISG-60) (IFN-induced 60 kDa protein) (IFI-60K) (Retinoic acid-induced gene G protein) (P60) (RIG-G)	2.07	1.88E-21
SAMD9L	Sterile α motif domain-containing protein 9-like (Fragment)	1.97	5.51E-06
DDX58	Antiviral innate immune response receptor RIG-I	1.88	3.16E-19
IFIH1	IFN-induced helicase C domain-containing protein 1	1.88	4.01E-15
PARP10	Poly [ADP-ribose] polymerase (PARP) (EC 2.4.2.-)	1.83	9.35E-07
SAMD9	Sterile α motif domain-containing protein 9 (Fragment)	1.79	1.76E-07
TRIM34	Tripartite motif-containing protein 34 (IFN-responsive finger protein 1) (RING finger protein 21)	1.71	2.07E-04
HERC6	Probable E3 ubiquitin-protein ligase HERC6	1.52	9.14E-14
REC8	Meiotic recombination protein REC8 homolog (Fragment)	1.51	1.81E-04
OAS1	2'-5' oligoadenylate synthetase 1 p49 isoform (Fragment)	1.51	2.69E-33
DTX3L	E3 ubiquitin-protein ligase DTX3L (EC 2.3.2.27) (B-lymphoma- and BAL-associated protein) (Protein deltex-3-like) (RING-type E3 ubiquitin transferase DTX3L; Rhysin-2)	1.46	1.44E-21
HELZ2	Helicase with zinc finger domain 2 (ATP-dependent helicase PRIC285) (transcriptional coactivator) (PPAR-α-interacting complex protein 285) (PPAR-γ DNA-binding domain-interacting protein 1) (PDIP1) (Peroxisomal proliferator-activated receptor A-interacting complex 285 kDa protein	1.38	3.78E-17
EIF2AK2	eIF2AK2 protein	1.36	3.59E-12
STAT1	Signal transducer and activator of transcription 1 (Fragment)	1.35	1.90E-28
OAS3	2'-5'-oligoadenylate synthetase 3, 100kDa, isoform CRA_a	1.35	3.29E-26
IFI16	Γ-IFN-inducible protein 16	1.35	4.30E-03
PLSCR1	Phospholipid scramblase (Fragment)	1.27	4.77E-13
IFIT2	IFN-induced protein with tetratricopeptide repeats 2	1.25	1.68E-02
SP110	Sp110 nuclear body protein (Fragment)	1.23	6.32E-08
CCL20	C-C motif chemokine 20 (Fragment)	1.20	3.03E-06
FGG	Fibrinogen γ chain	1.19	5.53E-03
DDX60L	Putative ATP-dependent RNA helicase DDX60 (EC 3.6.4.13)	1.13	1.68E-05

CFB	Complement factor B (Fragment)	1.12	7.26E-08
TRIM14	Tripartite motif-containing 14, isoform CRA_c	1.09	3.97E-11
IFIT5	IFN-induced protein with tetratricopeptide repeats 5 (IFIT-5) (Retinoic acid- and IFN-inducible 58 kDa protein) (P58)	1.09	3.90E-07
SAMHD1	Deoxynucleoside triphosphate triphosphohydrolase SAMHD1	1.08	5.81E-12
PHF11	PHD finger protein 11 (cDNA FLJ56933, highly similar to Homo sapiens PHD finger protein 11 (PHF11), transcript variant 1, mRNA)	1.07	2.63E-03
IFI35	IFN-induced 35 kDa protein (IFP 35) (Ifi-35)	1.06	1.54E-08
LAP3	Cytosol aminopeptidase (Fragment)	1.03	2.82E-13
CXCL5	C-X-C motif chemokine 5 (ENA-78(1-78)) (Epithelial-derived neutrophil-activating protein 78) (Small-inducible cytokine B5) [Cleaved into: ENA-78(8-78); ENA-78(9-78)]	0.96	3.86E-08
PARP14	Protein mono-ADP-ribosyltransferase PARP14	0.96	4.02E-08
HERC5	E3 ISG15--protein ligase HERC5 (HECT and RLD domain-containing E3 ubiquitin protein ligase 5) (Fragment)	0.94	3.70E-05
SP100	Nuclear autoantigen Sp-100 (Fragment)	0.94	1.23E-07
BCL2A1	Bcl-2-related protein A1 (Bcl-2-like protein 5) (Bcl2-L-5) (Hemopoietic-specific early response protein) (Protein BFL-1) (Protein GRS)	0.93	1.15E-02
IFITM3	IFN-induced transmembrane protein 3	0.91	1.75E-03
GBP3	Guanylate-binding protein 3	0.89	1.26E-02
USP18	Ubl carboxyl-terminal hydrolase 18 (EC 3.4.19.-) (43 kDa ISG15-specific protease) (hUBP43) (ISG15-specific-processing protease) (Ubl thioesterase 18)	0.86	1.21E-04
CP	CP protein (Ceruloplasmin (Ferroxidase)) (Ceruloplasmin (Ferroxidase), isoform CRA_a)	0.86	8.59E-04
CFH	Complement factor H	0.85	1.15E-02
PARP12	Poly (ADP-ribose) polymerase family, member 12, isoform CRA_b (Zinc finger CCCH type domain containing 1)	0.82	9.48E-05
STEAP1	Six transmembrane epithelial antigen of the prostate 1, isoform CRA_a	0.81	9.43E-03
EHF	ETS homologous factor (Fragment)	0.81	1.60E-02
PTGS2	Prostaglandin-endoperoxidase synthase 2 (EC 1.14.99.1) (Fragment)	0.80	1.47E-03
C1R	Complement C1r subcomponent	0.80	4.43E-07
SAT1	Diamine acetyltransferase 1	0.78	1.89E-06
BIVM-ERCC5	BIVM-ERCC5 readthrough (Fragment)	0.76	3.05E-02
C19orf66	Shiftless antiviral inhibitor of ribosomal frameshifting protein (SFL) (SHFL) (IFN-regulated antiviral protein) (IRAV) (Repressor of yield of DENV protein) (RyDEN)	0.76	1.03E-03
SNAP25	Synaptosomal-associated protein 25 (Fragment)	0.76	2.62E-03
CXCL8	Multifunctional fusion protein [Includes: Interleukin-8 (IL-8) (C-X-C motif chemokine 8)	0.75	1.17E-06
PDK4	Protein-serine/threonine kinase (EC 2.7.11.-)	0.75	1.29E-02
PNPT1	Polyribonucleotide nucleotidyltransferase 1, mitochondrial	0.74	9.53E-04
MMD	Monocyte to macrophage differentiation factor	0.72	2.08E-02
APOL6	Apolipoprotein L, 6 (Apolipoprotein L6) (cDNA FLJ38562 fis, clone HCHON2004002, similar to Apolipoprotein-L6)	0.71	3.42E-02
C1S	Complement C1s subcomponent	0.71	1.43E-06
CXCL2	C-X-C motif chemokine	0.71	2.37E-04
UBE2L6	Ubiquitin/ISG15-conjugating enzyme E2 L6 (Fragment)	0.71	3.07E-04
NUCB2	Nesfatin 1 (Fragment)	0.70	3.56E-02
APOL1	Apolipoprotein L, 1, isoform CRA_b	0.70	5.96E-03
PLA2G4A	Cytosolic phospholipase A2 (cPLA2) (Phospholipase A2 group IVA)	0.70	1.93E-02
PAPPA-AS1	Protein PAPPAS (DIPLA1 antisense RNA 1)	0.69	2.25E-02
TYMP	Thymidine phosphorylase isoform 2	0.66	1.33E-02
FGA	Fibrinogen α chain	0.66	9.95E-04

PTPN12	Tyrosine-protein phosphatase non-receptor type 12	0.66	1.56E-02
FILIP1	Filamin A interacting protein 1, isoform CRA_c (Filamin-A-interacting protein 1)	0.66	2.08E-02
ESF1	ESF1 homolog	0.64	3.36E-02
NCOA7	Nuclear receptor coactivator 7, isoform CRA_c	0.63	2.58E-03
CXCL3	C-X-C motif chemokine 3 (GRO-γ(1-73)) (Growth-regulated protein γ) (GRO-γ) (Macrophage inflammatory protein 2-β) (MIP2-β) [Cleaved into: GRO-γ(5-73)]	0.62	1.61E-02
B2M	B-2-microglobulin	0.61	1.17E-02
TDRD7	Tudor domain-containing protein 7 (PCTAIRE2-binding protein) (Tudor repeat associator with PCTAIRE-2) (Trap)	0.61	3.92E-03
C3	Complement C3 (Fragment)	0.61	1.47E-03
PSIP1	Alternative protein PSIP1	0.60	5.96E-03
KTN1	Kinectin 1 (Kinesin receptor), isoform CRA_a (Kinectin 1 (Kinesin receptor), isoform CRA_b)	0.59	3.36E-02
CXCL1	Growth-regulated α protein (C-X-C motif chemokine 1) (GRO-α(1-73)) (Melanoma growth stimulatory activity) (MGSA) (Neutrophil-activating protein 3) (NAP-3) [Cleaved into: GRO-α(4-73); GRO-α(5-73); GRO-α(6-73)]	0.59	2.00E-03
TCIM	Transcriptional and immune response regulator (Thyroid cancer protein 1) (TC-1)	0.58	5.53E-03
ADAR	Double-stranded RNA-specific adenosine deaminase (fragment)	0.57	1.92E-05
DKK1	Dickkopf-like protein 1	0.57	1.24E-03
KYNU	Kynureninase (Fragment)	0.57	2.98E-02
AREG	Amphiregulin	0.57	2.09E-03
LGALS3BP	Lectin galactoside-binding soluble 3 binding protein isoform 1 (CRA_d) (Fragment)	0.55	1.25E-02
CDH1	Cadherin-1	0.55	2.88E-03
STAT2	Signal transducer and activator of transcription 2	0.55	7.66E-03
HIF1A	Hypoxia-inducible factor 1, α subunit (Basic helix-loop-helix transcription factor), isoform CRA_a	0.54	3.82E-02
TRIM25	E3 ubiquitin/ISG15 ligase TRIM25	0.53	7.01E-04
HSP90B1	Endoplasmin	0.48	3.44E-02
EDN1	Endothelin-1 (Preproendothelin-1) (PPET1) [Cleaved into: Endothelin-1 (ET-1); Big endothelin-1]	0.45	2.50E-02
**Downregulated 20 genes in SARS-CoV-2-infected A549 cells**			
SQSTM1	Sequestosome-1	-0.46	2.50E-02
NPTX1	Neuronal pentraxin-1 (NP1) (Neuronal pentraxin I) (NP-I)	-0.51	2.25E-02
AHNAK2	Protein AHNAK2	-0.51	3.19E-03
NEU1	Sialidase-1	-0.52	1.24E-02
WDR81	WD repeat-containing protein 81	-0.53	2.50E-02
FAM102A	Protein FAM102A (Early estrogen-induced gene 1 protein)	-0.57	2.34E-03
DANCR	Uncharacterized protein DANCR (Anti-differentiation ncRNA protein) (Small nucleolar RNA host gene protein 13)	-0.59	1.84E-02
NT5DC2	5'-nucleotidase domain-containing protein 2 (Fragment)	-0.59	1.23E-02
MAP3K14	Mitogen-activated protein kinase kinase kinase 14 (Fragment)	-0.59	1.44E-02
KLHL21	Kelch-like protein 21	-0.61	5.58E-03
LOC284454	/ (ncRNA)	-0.64	3.92E-02
SYNE1	Nesprin-1	-0.68	2.34E-03
COL1A1	Collagen, type I, α 1, isoform CRA_a	-0.73	1.05E-03
OSGIN1	Oxidative stress-induced growth inhibitor 1 (Fragment)	-0.74	6.80E-04
RAP1GAP	Rap1 GTPase-activating protein 1	-0.74	6.04E-05
NPIPB5	Nuclear pore complex-interacting protein family member B5	-0.83	1.06E-02
UAP1L1	UDP-N-acetylhexosamine pyrophosphorylase-like protein 1	-0.84	4.55E-04
NUPR1	Nuclear protein 1	-0.99	5.96E-03
NECAB2	N-terminal EF-hand calcium-binding protein 2 (Fragment)	-1.12	2.50E-02
KRT4	Keratin, type II cytoskeletal 4	-1.23	1.84E-04

Table S2A. **Properties of the 36 DEGs that define the antiviral gene signature derived from the transciptome of SARS-CoV-2-infected A549 cells**

Index	Gene name	Protein name	Log_2 f Change	Database	GO Annotation	GO number
**UPREGULATED GENES THAT SHOULD BE UPREGULATED BY SMALL MOLECULES**						
**1**	IFI6	IFNα-inducible protein 6	4.27	GO:BP	type I IFN signaling pathway	GO:0060337
					defense response to virus	GO:0051607
					cellular response to type I IFN	GO:0071357
					response to type I IFN	GO:0034340
**2**	IRF7	IFN regulatory factor 7, isoform CRA_a	3.13	GO:BP	cellular response to IFN-γ	GO:0071346
					type I IFN signaling pathway	GO:0060337
					cellular response to type I IFN	GO:0071357
					response to type I IFN	GO:0034340
					response to IFN-γ	GO:0034341
					defense response to virus	GO:0051607
					regulation of response to cytokine stimulus	GO:0060759
					IFN-γ-mediated signaling pathway	GO:0060333
**3**	DDX60	ATP-dependent RNA helicase DDX60	2.42	GO:BP	defense response to virus	GO:0051607
**4**	PARP9	Protein mono-ADP-ribosyltransferase PARP9 (Fragment)	2.13	GO:BP	regulation of response to cytokine stimulus	GO:0060759
					cellular response to IFN-γ	GO:0071346
					IFN-γ-mediated signaling pathway	GO:0060333
					response to IFN-γ	GO:0034341
					defense response to virus	GO:0051607
**5**	IRF9	IFN regulatory factor 9	2.11	GO:BP	response to type I IFN	GO:0034340
					cellular response to IFN-γ	GO:0071346
					response to IFN-γ	GO:0034341
					IFN-γ-mediated signaling pathway	GO:0060333
					cellular response to type I IFN	GO:0071357
					type I IFN signaling pathway	GO:0060337
					defense response to virus	GO:0051607
**6**	IFIT3	IFN-induced protein with tetratricopeptide repeats 3	2.07	GO:BP	cellular response to type I IFN	GO:0071357
					defense response to virus	GO:0051607
					type I IFN signaling pathway	GO:0060337
					response to type I IFN	GO:0034340
**7**	DDX58	Antiviral innate immune response receptor RIG-I	1.88	GO:BP	defense response to virus	GO:0051607
					regulation of response to cytokine stimulus	GO:0060759
**8**	IFIH1	IFN-induced helicase C domain-containing protein 1	1.88	GO:BP	defense response to virus	GO:0051607
					regulation of response to cytokine stimulus	GO:0060759
**9**	TRIM34	Tripartite motif-containing protein 34 (IFN-responsive finger protein 1) (RING finger protein 21)	1.71	GO:BP	defense response to virus	GO:0051607
					response to IFN-γ	GO:0034341
					IFN-γ-mediated signaling pathway	GO:0060333
					cellular response to IFN-γ	GO:0071346
**10**	DTX3L	E3 ubiquitin-protein ligase DTX3L	1.46	GO:BP	defense response to virus	GO:0051607
**11**	STAT1	Signal transducer and activator of transcription 1 (Fragment)	1.35	GO:BP	regulation of response to cytokine stimulus	GO:0060759
					type I IFN signaling pathway	GO:0060337
					defense response to virus	GO:0051607
					cellular response to type I IFN	GO:0071357
					negative regulation of viral process	GO:0048525

					response to type I IFN	GO:0034340
					IFN-γ-mediated signaling pathway	GO:0060333
					response to IFN-γ	GO:0034341
					regulation of symbiosis, encompassing mutualism through parasitism	GO:0043903
					negative regulation of multi-organism process	GO:0043901
					cellular response to IFN-γ	GO:0071346
					regulation of viral process	GO:0050792
**12**	IFIT2	IFN-induced protein with tetratricopeptide repeats 2	1.25	GO:BP	cellular response to type I IFN	GO:0071357
					defense response to virus	GO:0051607
					type I IFN signaling pathway	GO:0060337
					response to type I IFN	GO:0034340
**13**	CCL20	C-C motif chemokine 20 (Fragment)	1.2	GO:BP	response to IFN-γ	GO:0034341
					cellular response to IFN-γ	GO:0071346
**14**	TRIM14	Tripartite motif-containing 14, isoform CRA_c	1.09	GO:BP	regulation of viral process	GO:0050792
					regulation of symbiosis, encompassing mutualism through parasitism	GO:0043903
					negative regulation of multi-organism process	GO:0043901
					negative regulation of viral process	GO:0048525
**15**	SAMHD1	Deoxynucleoside triphosphate triphosphohydrolase SAMHD1	1.08	GO:BP	cellular response to type I IFN	GO:0071357
					negative regulation of multi-organism process	GO:0043901
					regulation of response to cytokine stimulus	GO:0060759
					response to type I IFN	GO:0034340
					defense response to virus	GO:0051607
					type I IFN signaling pathway	GO:0060337
**16**	IFI35	IFN-induced 35 kDa protein (IFP 35) (Ifi-35)	1.06	GO:BP	type I IFN signaling pathway	GO:0060337
					cellular response to type I IFN	GO:0071357
					response to type I IFN	GO:0034340
**17**	PARP14	Protein mono-ADP-ribosyltransferase PARP14	0.96	GO:BP	regulation of response to cytokine stimulus	GO:0060759
					IFN-γ-mediated signaling pathway	GO:0060333
					cellular response to IFN-γ	GO:0071346
					response to IFN-γ	GO:0034341
					negative regulation of multi-organism process	GO:0043901
**18**	HERC5	E3 ISG15--protein ligase HERC5 (Fragment)	0.94	GO:BP	defense response to virus	GO:0051607
**19**	SP100	Nuclear autoantigen Sp-100 (Fragment)	0.94	GO:BP	cellular response to type I IFN	GO:0071357
					type I IFN signaling pathway	GO:0060337
					response to type I IFN	GO:0034340
					response to IFN-γ	GO:0034341
					IFN-γ-mediated signaling pathway	GO:0060333
					cellular response to IFN-γ	GO:0071346
**20**	GBP3	Guanylate-binding protein 3	0.89	GO:BP	response to IFN-γ	GO:0034341
					cellular response to IFN-γ	GO:0071346
					defense response to virus	GO:0051607
**21**	USP18	Ubl carboxyl-terminal hydrolase 18	0.86	GO:BP	type I IFN signaling pathway	GO:0060337
					cellular response to type I IFN	GO:0071357
					response to type I IFN	GO:0034340
					regulation of response to cytokine stimulus	GO:0060759
**22**	B2M	B-2-microglobulin	0.61	GO:BP	response to IFN-γ	GO:0034341
					cellular response to IFN-γ	GO:0071346
					IFN-γ-mediated signaling pathway	GO:0060333
**23**	KYNU	Kynureninase (Fragment)	0.57	GO:BP	response to IFN-γ	GO:0034341
**24**	STAT2	Signal transducer and activator of transcription 2	0.55	GO:BP	defense response to virus	GO:0051607
					response to type I IFN	GO:0034340
					cellular response to type I IFN	GO:0071357

					type I IFN signaling pathway	GO:0060337
**25**	TRIM25	E3 ubiquitin/ISG15 ligase TRIM25	0.53	GO:BP	cellular response to IFN-γ	GO:0071346
					regulation of viral life cycle	GO:1903900
					response to IFN-γ	GO:0034341
					negative regulation of viral life cycle	GO:1903901
					IFN-γ-mediated signaling pathway	GO:0060333
					regulation of viral process	GO:0050792
					negative regulation of multi-organism process	GO:0043901
					defense response to virus	GO:0051607
					negative regulation of viral process	GO:0048525
					regulation of symbiosis, encompassing mutualism through parasitism	GO:0043903
**26**	EDN1	Endothelin-1 (Preproendothelin-1) (PPET1)	0.45	GO:BP	regulation of response to cytokine stimulus	GO:0060759
					response to IFN-γ	GO:0034341
					cellular response to IFN-γ	GO:0071346
**DOWREGULATED GENES THAT SHOULD BE UPREGULATED BY SMALL MOLECULES**						
**1**	SQSTM1	Sequestosome-1	-0.46	GO:CC	supramolecular complex	GO:0099080
					intracellular vesicle	GO:0097708
					supramolecular fiber	GO:0099512
					supramolecular polymer	GO:0099081
					cytoplasmic vesicle	GO:0031410
					myofibril	GO:0030016
					sarcomere	GO:0030017
					contractile fiber	GO:0043292
					amphisome	GO:0044753
					P-body	GO:0000932
					autophagosome	GO:0005776
					vesicle	GO:0031982
**2**	AHNAK2	Protein AHNAK2	-0.51	GO:CC	supramolecular complex	GO:0099080
					intracellular vesicle	GO:0097708
					cytoplasmic vesicle	GO:0031410
					sarcomere	GO:0030017
					supramolecular polymer	GO:0099081
					supramolecular fiber	GO:0099512
					myofibril	GO:0030016
					contractile fiber	GO:0043292
					vesicle	GO:0031982
**3**	NPTX1	Neuronal pentraxin-1 (NP1) (Neuronal pentraxin I) (NP-I)	-0.51	GO:CC	cytoplasmic vesicle	GO:0031410
					intracellular vesicle	GO:0097708
					vesicle	GO:0031982
**4**	NEU1	Sialidase-1	-0.52	GO:CC	cytoplasmic vesicle	GO:0031410
					intracellular vesicle	GO:0097708
					vesicle	GO:0031982
**5**	WDR81	WD repeat-containing protein 81	-0.53	GO:CC	cytoplasmic vesicle	GO:0031410
					vesicle	GO:0031982
					autophagosome	GO:0005776
					intracellular vesicle	GO:0097708
**6**	KLHL21	Kelch-like protein 21	-0.61	GO:CC	supramolecular polymer	GO:0099081
					cytoplasmic vesicle	GO:0031410
					intracellular vesicle	GO:0097708
					supramolecular complex	GO:0099080
					vesicle	GO:0031982
					supramolecular fiber	GO:0099512
**7**	SYNE1	Nesprin-1	-0.68	GO:CC	P-body	GO:0000932
					myofibril	GO:0030016

					contractile fiber	GO:0043292
					supramolecular polymer	GO:0099081
					sarcomere	GO:0030017
					supramolecular complex	GO:0099080
					supramolecular fiber	GO:0099512
**8**	COL1A1	Collagen, type I, *α* 1, isoform CRA_a	-0.73	GO:CC	vesicle	GO:0031982
					supramolecular fiber	GO:0099512
					supramolecular polymer	GO:0099081
					cytoplasmic vesicle	GO:0031410
					intracellular vesicle	GO:0097708
					supramolecular complex	GO:0099080
					collagen type I trimer	GO:0005584
**9**	RAP1GAP	Rap1 GTPase-activating protein 1	-0.74	GO:CC	cytoplasmic vesicle	GO:0031410
					vesicle	GO:0031982
					intracellular vesicle	GO:0097708
**10**	KRT4	Keratin, type II cytoskeletal 4	-1.23	GO:CC	supramolecular fiber	GO:0099512
					supramolecular polymer	GO:0099081
					supramolecular complex	GO:0099080

** Showing enrichment result from Gene Ontology Biological Process (GO:BP) and Cellular Component (GO:CC) databases with term size < 300 genes and overlap size > 10 genes.*

Table S2B. Properties of the 17 DEGs that define the anti-cytokine signature derived from the transcriptome of SARS-CoV-2-infected A549-ACE2 cells

Index	Gene name	Protein name	Log_2 Fold Change	$P_{adjusted}$	Database	GO Annotation	GO number
**1**	EGR1	Early growth response protein	6.62	0.00E+00	GO:BP	cytokine-mediated signaling pathway	GO:0019221
						cellular response to cytokine stimulus	GO:0071345
**2**	IFNB1	Interferon β	5.76	1.15E-28	GO:BP	cytokine-mediated signaling pathway	GO:0019221
						cellular response to cytokine stimulus	GO:0071345
						regulation of response to external stimulus	GO:0032101
						positive regulation of immune system process	GO:0002684
**3**	CXCL2	C-X-C motif chemokine	5.27	0.00E+00	GO:BP	cytokine-mediated signaling pathway	GO:0019221
						cellular response to cytokine stimulus	GO:0071345
**4**	NFKBIA	NFκB inhibitor α	5.23	0.00E+00	GO:BP	cytokine-mediated signaling pathway	GO:0019221
						cellular response to cytokine stimulus	GO:0071345
						regulation of response to external stimulus	GO:0032101
**5**	SELE	E-selectin	4.85	1.59E-25	GO:BP	regulation of response to external stimulus	GO:0032101
						positive regulation of immune system process	GO:0002684
**6**	IL8	Multifunctional fusion protein [Includes: Interleukin-8	4.85	0.00E+00	GO:BP	cytokine-mediated signaling pathway	GO:0019221
						cellular response to cytokine stimulus	GO:0071345
						regulation of response to external stimulus	GO:0032101
						positive regulation of immune system process	GO:0002684
**7**	IRF7	Interferon regulatory factor 7	4.81	2.69E-97	GO:BP	cytokine-mediated signaling pathway	GO:0019221
						cellular response to cytokine stimulus	GO:0071345
						regulation of response to external stimulus	GO:0032101
						positive regulation of immune system process	GO:0002684
**8**	IFITM1	Interferon induced transmembrane protein 1	4.58	3.21E-47	GO:BP	cytokine-mediated signaling pathway	GO:0019221
						cellular response to cytokine stimulus	GO:0071345
**9**	NFIL3	Nuclear factor interleukin-3-	4.22	0.00E+00	GO:BP	cellular response to cytokine stimulus	GO:0071345

		regulated protein					
**10**	TNF	Tumor necrosis factor	4.16	3.70E-29	GO:BP	cytokine-mediated signaling pathway	GO:0019221
						cellular response to cytokine stimulus	GO:0071345
						regulation of response to external stimulus	GO:0032101
						positive regulation of immune system process	GO:0002684
**11**	CXCL3	C-X-C motif chemokine 3	4.03	0.00E+00	GO:BP	cytokine-mediated signaling pathway	GO:0019221
						cellular response to cytokine stimulus	GO:0071345
**12**	SOCS1	Suppressor of cytokine signaling 1	4.02	1.48E-16	GO:BP	cytokine-mediated signaling pathway	GO:0019221
						cellular response to cytokine stimulus	GO:0071345
						regulation of response to external stimulus	GO:0032101
						positive regulation of immune system process	GO:0002684
**13**	CD274	Programmed cell death 1 ligand 1	4.01	1.20E-46	GO:BP	positive regulation of immune system process	GO:0002684
**14**	IL20RB	Interleukin-20 receptor subunit β	3.79	1.15E-34	GO:BP	cytokine-mediated signaling pathway	GO:0019221
						cellular response to cytokine stimulus	GO:0071345
						regulation of response to external stimulus	GO:0032101
**15**	CCL20	C-C motif chemokine 20	3.77	0.00E+00	GO:BP	cytokine-mediated signaling pathway	GO:0019221
						cellular response to cytokine stimulus	GO:0071345
						positive regulation of immune system process	GO:0002684
**16**	CCR6	C-C chemokine receptor type 6	3.66	1.60E-16	GO:BP	cytokine-mediated signaling pathway	GO:0019221
						cellular response to cytokine stimulus	GO:0071345
						regulation of response to external stimulus	GO:0032101
						positive regulation of immune system process	GO:0002684
**17**	HLA-F	HLA-F	3.49	3.59E-86	GO:BP	cytokine-mediated signaling pathway	GO:0019221
						cellular response to cytokine stimulus	GO:0071345
						regulation of response to external stimulus	GO:0032101
						positive regulation of immune system process	GO:0002684

** Gene name: gene symbol of the DEGs. Protein name: protein name of corresponding genes from UniProt[1] (Genes are ordered by log_2 fold change in descending order. see STAR*Methods for the definition of adjusted P. Showing enrichment results from GO Biological Process database with term size < 1,200 genes and overlap size > 10 genes. Log_2 Fold Change: log2 transformation of the fold change in gene expression level after viral infection;* ***Database****: Gene ontology (GO) database used. BP refers to biological process, CC refers to cellular component;* ***Term****: name of the gene set in the GO database;* ***GO number****: the index of the GO term. * Genes are ordered by descending log_2 Fold Change.*

Table S3A. 263 compounds with potential antiviral activity against SARS-CoV-2 infected A549 cells and corresponding CMap scores (all > 90)

Index	Compound name	CMap ID	CMap score	Mechanism of Action (MOA)
1	Salmeterol	BRD-A01320529	100.00	Adrenergic receptor agonist
2	Avrainvillamide-analog-5	BRD-A70731303	99.99	nucleophosmin inhibitor
3	Terbutaline	BRD-A50157456	99.98	Adrenergic receptor agonist
4	Oxybutynin	BRD-A65013509	99.98	Acetylcholine receptor antagonist
5	Leflunomide	BRD-K78692225	99.98	Dihydroorotate dehydrogenase inhibitor, PDGFR receptor inhibitor
6	GS-39783	BRD-K75478907	99.98	GABA receptor modulator
7	nTZDpa	BRD-K54708045	99.98	PPAR receptor agonist
8	n-arachidonyl-GABA	BRD-K06024458	99.98	cannabinoid receptor agonist
9	Salicin	BRD-K64614248	99.98	Anti-inflammatory
10	SR-27897	BRD-K35629949	99.98	CCK receptor antagonist
11	Thiotepa	BRD-K09631521	99.98	Cytochrome P450 inhibitor
12	Brazilin	BRD-A83326220	99.98	Nitric oxide production inhibitor
13	Stavudine	BRD-K93880783	99.98	DNA directed DNA polymerase inhibitor, Reverse transcriptase inhibitor
14	Oxiconazole	BRD-K23369905	99.98	Bacterial cell wall synthesis inhibitor
15	Mirtazapine	BRD-A64977602	99.98	Adrenergic receptor antagonist, Serotonin receptor antagonist
16	Liothyronine	BRD-K89152108	99.98	Thyroid hormone stimulant
17	Bergenin	BRD-A15034104	99.98	Interleukin inhibitor
18	Chlorpromazine	BRD-K89997465	99.98	Dopamine receptor antagonist
19	Fludarabine	BRD-K66788707	99.96	DNA synthesis inhibitor, DNA repair enzyme inhibitor, Purine antagonist
20	Iodophenpropit	BRD-K51918615	99.96	Histamine receptor antagonist
21	SA-94315	BRD-K20197062	99.96	Caspase inhibitor
22	L-733060	BRD-K15791587	99.96	Tachykinin antagonist
23	Tetrahydrobiopterin	BRD-A67605442	99.96	Nitric oxide (NO) stimulant, NO synthase stimulant, Phenylalanine 4-hydroxylase stimulant
24	Duloxetine	BRD-K71103788	99.96	Serotonin and norepinephrine reuptake inhibitor
25	PSB-36	BRD-A70407468	99.96	Adenosine receptor antagonist
26	CGP-7930	BRD-K65786282	99.95	GABA receptor positive allosteric modulator
27	Ochratoxin-a	BRD-K39944607	99.95	Phenylalanyl tRNA synthetase inhibitor
28	Alfacalcidol	BRD-K93433262	99.94	Vitamin D receptor agonist
29	Molsidomine	BRD-K35531059	99.94	Guanylyl cyclase activator
30	NGB-2904	BRD-K05181084	99.94	Dopamine receptor antagonist
31	BH3I-1	BRD-A38913120	99.93	BCL inhibitor
32	Isotretinoin	BRD-K76723084	99.93	Retinoid receptor agonist
33	Lonidamine	BRD-K96670504	99.92	Glucokinase inhibitor
34	L-741626	BRD-K05181463	99.91	Dopamine receptor antagonist
35	PG-9	BRD-A70268693	99.91	Acetylcholine receptor agonist
36	GSK-1059615	BRD-K06750613	99.90	PI3K inhibitor
37	Chenodeoxycholic-acid	BRD-K18135438	99.90	11-β-HSD1 inhibitor, FXR agonist
38	Edrophonium	BRD-K81128206	99.89	Acetylcholinesterase inhibitor
39	Nilotinib	BRD-K81528515	99.89	ABL inhibitor, BCR-ABL kinase inhibitor
40	5-nonyloxytryptamine	BRD-K08219523	99.88	Serotonin receptor agonist
41	Tribenoside	BRD-A60294240	99.88	Anti-inflammatory, Capillary stabilizing agent
42	Meclozine	BRD-A50311610	99.85	CAR agonist
43	Azathioprine	BRD-K32821942	99.85	Dehydrogenase inhibitor
44	HDAC3-selective	BRD-K29313308	99.84	HDAC inhibitor
45	MRS-1845	BRD-A32949107	99.84	Calcium channel blocker
46	KU-55933	BRD-K25311561	99.84	ATM kinase inhibitor
47	Arecaidine	BRD-K63792901	99.84	Acetylcholine receptor agonist
48	Cortisone	BRD-A54487287	99.83	Glucocorticoid receptor agonist
49	Torin-1	BRD-K40175214	99.80	MTOR inhibitor, PI3K inhibitor
50	ENMD-2076	BRD-K68488863	99.79	FLT3 inhibitor, VEGFR inhibitor, Aurora kinase inhibitor
51	Aspirin	BRD-K11433652	99.79	Cyclooxygenase inhibitor
52	AS-703026	BRD-K89014967	99.74	MEK inhibitor
53	Razoxane	BRD-K07265709	99.74	Chelating agent, Topoisomerase inhibitor
54	Ibuprofen	BRD-A17655518	99.72	Cyclooxygenase inhibitor, NFkB pathway inhibitor

**55**	Demeclocycline	BRD-A75368507	99.71	Bacterial 30S ribosomal subunit inhibitor
**56**	KU-0063794	BRD-K67566344	99.71	MTOR inhibitor
**57**	Desoxycorticosterone	BRD-A75402480	99.69	Mineralocorticoid receptor agonist
**58**	Imipramine	BRD-K38436528	99.65	Norepinephrine and Serotonin transporter inhibitor
**59**	AS-605240	BRD-K41895714	99.63	PI3K inhibitor
**60**	OSI-027	BRD-K94294671	99.62	MTOR inhibitor
**61**	PD-0325901	BRD-K49865102	99.59	MEK inhibitor, MAPK inhibitor, Protein kinase inhibitor
**62**	PKCβ-inhibitor	BRD-K89687904	99.59	PKC inhibitor
**63**	Fostamatinib	BRD-K20285085	99.58	SYK inhibitor
**64**	Semaxanib	BRD-K63504947	99.57	VEGFR inhibitor
**65**	SB-216641	BRD-K30867024	99.57	Serotonin receptor antagonist
**66**	Rescinnamine	BRD-K52930707	99.53	ACE inhibitor
**67**	Tolazoline	BRD-K46211610	99.53	Adrenergic receptor antagonist
**68**	Dactolisib	BRD-K12184916	99.51	MTOR inhibitor, PI3K inhibitor, Protein kinase inhibitor
**69**	GSK-1904529A	BRD-K04833372	99.50	IGF-1 inhibitor, IGF-1R inhibitor, Insulin receptor ligand
**70**	ML-9	BRD-K68402494	99.44	Myosin light chain kinase inhibitor
**71**	UNC-0321	BRD-K74236984	99.42	Histone lysine methyltransferase inhibitor
**72**	Pizotifen	BRD-K75958195	99.41	Serotonin receptor antagonist
**73**	Pterostilbene	BRD-K92870997	99.40	Cyclooxygenase inhibitor, PPAR receptor agonist
**74**	VER-155008	BRD-K32330832	99.39	HSP inhibitor
**75**	PIK-90	BRD-K99818283	99.35	PI3K inhibitor
**76**	Panobinostat	BRD-K02130563	99.34	HDAC inhibitor
**77**	Oleoylethanolamide	BRD-K66956375	99.31	Cannabinoid receptor agonist, Glucose dependent insulinotropic receptor agonist, Potassium channel blocker, PPAR receptor agonist
**78**	TC-2559	BRD-K67352070	99.30	Acetylcholine receptor agonist
**79**	Mosapride	BRD-A39052811	99.26	Serotonin receptor agonist
**80**	Dapsone	BRD-K62363391	99.21	Bacterial antifolate
**81**	BAY-36-7620	BRD-K54704028	99.20	Glutamate receptor antagonist
**82**	Emetine	BRD-A25687296	99.20	Protein synthesis inhibitor
**83**	Arecaidine	BRD-K23922020	99.19	Acetylcholine receptor agonist
**84**	Apicidin	BRD-K64606589	99.18	HDAC inhibitor
**85**	Niacin	BRD-K61993165	99.16	NAD precursor with lipid lowering effect, vitamin B
**86**	Entinostat	BRD-K77908580	99.16	HDAC inhibitor
**87**	Elesclomol	BRD-K82135108	99.15	Oxidative stress inducer
**88**	BMY-14802	BRD-A15435692	99.14	Sigma receptor antagonist
**89**	Proxymetacaine	BRD-K79116891	99.14	Sodium channel blocker
**90**	Zamifenacin	BRD-K80451230	99.14	Acetylcholine receptor antagonist
**91**	Anandamide	BRD-K78280988	99.12	Cannabinoid receptor agonist
**92**	Temsirolimus	BRD-A62025033	99.10	MTOR inhibitor
**93**	Desmethylclozapine	BRD-K10042277	99.07	Acetylcholine receptor agonist
**94**	QL-XII-47	BRD-U86922168	99.03	BTK inhibitor, Cytoplasmic tyrosine protein kinase BMX inhibitor
**95**	PI-103	BRD-K67868012	99.03	MTOR inhibitor, PI3K inhibitor
**96**	Eugenol	BRD-K32977963	99.01	Androgen receptor antagonist
**97**	SKF-81297	BRD-A09828896	98.98	Dopamine receptor agonist
**98**	2-aminopurine	BRD-K35128472	98.97	Serine/threonine kinase inhibitor
**99**	GBR-12783	BRD-K92015269	98.97	Dopamine uptake inhibitor
**100**	Mephenytoin	BRD-A83937277	98.96	Hydantoin antiepileptic
**101**	Terfenadine	BRD-A06352418	98.89	Histamine receptor antagonist
**102**	KIN001-127	BRD-A29901043	98.89	ITK inhibitor
**103**	GBR-12935	BRD-K50135270	98.88	Dopamine uptake inhibitor
**104**	Flavanone	BRD-A07824748	98.74	11-β-HSD1 inhibitor
**105**	VU-0366037-2	BRD-K39823328	98.73	Glutamate receptor modulator
**106**	Ioxaglic-acid	BRD-K79124250	98.68	Radiopaque medium
**107**	FR-122047	BRD-K30990140	98.65	Cyclooxygenase inhibitor
**108**	WZ-3146	BRD-K73293050	98.61	EGFR inhibitor
**109**	Droxinostat	BRD-K11558771	98.60	HDAC inhibitor
**110**	Cimaterol	BRD-A65440446	98.56	Adrenergic receptor agonist
**111**	SCH-23390	BRD-K45435259	98.48	Dopamine receptor antagonist
**112**	Ribavirin	BRD-A96255180	98.45	Antiviral
**113**	Mycophenolate-mofetil	BRD-K92428153	98.41	Dehydrogenase inhibitor, Hydroxycarboxylic acid receptor agonist, Immunosuppressant, Inosine monophosphate dehydrogenase inhibitor, Inositol monophosphatase inhibitor
**114**	Linsitinib	BRD-K08589866	98.37	IGF-1 inhibitor
**115**	LY-288513	BRD-K24675965	98.28	CCK receptor antagonist
**116**	Wiskostatin	BRD-A18579359	98.25	Neural Wiskott-Aldrich syndrome protein inhibitor

**117**	AG-879	BRD-K59469039	98.23	Angiogenesis inhibitor, Tyrosine kinase inhibitor, VEGFR inhibitor
**118**	BIX-01338	BRD-K26863634	98.21	Histone lysine methyltransferase inhibitor
**119**	Arcyriaflavin-a	BRD-K72726508	98.20	CDK inhibitor
**120**	AY-9944	BRD-K03642198	98.15	Hedgehog pathway modulator
**121**	NU-7026	BRD-K09537769	98.15	DNA dependent protein kinase inhibitor, MTOR inhibitor, PI3K inhibitor
**122**	GW-9662	BRD-K93258693	98.08	PPAR receptor antagonist
**123**	APHA-compound-8	BRD-K74733595	98.07	HDAC inhibitor
**124**	Mefenamic-acid	BRD-K92778217	98.00	Cyclooxygenase inhibitor
**125**	Heliomycin	BRD-K64517075	98.00	ATP synthase inhibitor
**126**	PP-30	BRD-K30677119	97.98	RAF inhibitor
**127**	NVP-TAE684	BRD-K50140147	97.96	ALK inhibitor
**128**	Ropivacaine	BRD-K50938786	97.64	Sodium channel blocker
**129**	MK-5108	BRD-K53665955	97.59	Aurora kinase inhibitor
**130**	Ciclacillin	BRD-K89046952	97.54	Bacterial cell wall synthesis inhibitor
**131**	Sulfafurazole	BRD-K50859149	97.43	Bacterial antifolate
**132**	Dephostatin	BRD-K60274257	97.43	Tyrosine phosphatase inhibitor
**133**	Entacapone	BRD-K83636919	97.31	Catechol O methyltransferase inhibitor
**134**	Oxfendazole	BRD-A33447119	97.21	Anthelmintic
**135**	Rottlerin	BRD-K03816923	97.11	MAP kinase inhibitor, Protein kinase inhibitor
**136**	Desipramine	BRD-K60762818	97.10	Tricyclic antidepressant
**137**	Perospirone	BRD-K85503079	97.10	Dopamine and serotonin receptors' antagonist
**138**	Pimozide	BRD-K01292756	97.10	Dopamine receptor antagonist
**139**	Ceforanide	BRD-K37848908	97.00	Penicillin binding protein inhibitor
**140**	Equilin	BRD-K04046242	96.99	Estrogen receptor agonist
**141**	SB-590885	BRD-K01253243	96.95	RAF inhibitor
**142**	LY-2140023	BRD-K49519144	96.93	Glutamate receptor agonist
**143**	Glipizide	BRD-K12219985	96.92	Sulfonylurea
**144**	Moracizine	BRD-K21548250	96.91	Sodium channel blocker
**145**	Kavain	BRD-A75455249	96.82	Calcium channel modulator, Sodium channel blocker
**146**	Wortmannin	BRD-A11678676	96.81	PI3K inhibitor
**147**	Decitabine	BRD-K79254416	96.67	DNA methyltransferase inhibitor
**148**	Metformin	BRD-K79602928	96.61	Insulin sensitizer
**149**	Eicosatrienoic-acid	BRD-K63913457	96.58	Vasodilator
**150**	Raloxifene	BRD-K63828191	96.58	Estrogen receptor antagonist, Selective estrogen receptor modulator (SERM)
**151**	Ezetimibe	BRD-A41519720	96.57	Niemann-Pick C1-like 1 protein antagonist, Cholesterol inhibitor
**152**	NBI-27914	BRD-K61177364	96.54	CRF receptor antagonist
**153**	RS-67333	BRD-K46142322	96.52	Serotonin receptor partial agonist
**154**	BMS-191011	BRD-K95609758	96.46	Potassium channel activator
**155**	H-7	BRD-A55756846	96.39	PKA inhibitor
**156**	VU-0404997-2	BRD-A34208323	96.39	Glutamate receptor modulator
**157**	Cisapride	BRD-K06895174	96.23	Serotonin receptor agonist
**158**	Y-134	BRD-K94832621	96.20	Estrogen receptor antagonist
**159**	Metrizamide	BRD-A45543382	95.98	Radiopaque medium
**160**	Dydrogesterone	BRD-K68620903	95.94	Progesterone receptor agonist
**161**	Altrenogest	BRD-A27554692	95.93	Progestogen hormone
**162**	Homosalate	BRD-A34751532	95.90	HSP inducer
**163**	Bosutinib	BRD-K99964838	95.89	ABL inhibitor, BCR-ABL kinase inhibitor, SRC inhibitor
**164**	Puromycin	BRD-A28970875	95.88	Protein synthesis inhibitor
**165**	Methimazole	BRD-K54416256	95.71	Antithyroid
**166**	ALW-II-38-3	BRD-K68191783	95.60	Ephrin inhibitor
**167**	SN-38	BRD-A36630025	95.51	Topoisomerase inhibitor
**168**	Ipratropium	BRD-A05352148	95.50	Acetylcholine receptor antagonist
**169**	TGX-221	BRD-A41692738	95.47	PI3K inhibitor
**170**	Homoharringtonine	BRD-K76674262	95.43	Protein synthesis inhibitor
**171**	Metergoline	BRD-A30435184	95.42	Dopamine receptor agonist, Serotonin receptor antagonist
**172**	WZ-4-145	BRD-U25771771	95.41	EGFR inhibitor
**173**	Mercaptopurine	BRD-K91601245	95.33	Immunosuppressant, Protein synthesis inhibitor, Purine antagonist
**174**	Calmidazolium	BRD-A98283014	95.28	Calcium channel blocker, Calmodulin antagonist
**175**	Mesna	BRD-M40783228	95.21	Antioxidant
**176**	SDZ-205-557	BRD-K15868788	95.20	Serotonin receptor antagonist
**177**	Procyclidine	BRD-A31800922	95.16	Acetylcholine receptor antagonist
**178**	Amiodarone	BRD-K17561142	95.15	Potassium channel blocker

**179**	Midodrine	BRD-A79981887	95.14	Adrenergic receptor agonist
**180**	Mepireserpate	BRD-A71765365	95.11	Catecholamine depleting sympatholytic
**181**	SA-792728	BRD-K20755323	95.06	Sphingosine kinase inhibitor
**182**	Brompheniramine	BRD-A68723818	94.90	Histamine receptor antagonist
**183**	Sumatriptan	BRD-K50938287	94.83	Serotonin receptor agonist
**184**	Gemcitabine	BRD-K15108141	94.82	Ribonucleotide reductase inhibitor
**185**	JAK3-Inhibitor-II	BRD-K52850071	94.81	JAK inhibitor
**186**	CHEMBL-374350	BRD-K59962020	94.79	NFkB pathway inhibitor
**187**	Vorinostat	BRD-K81418486	94.70	HDAC inhibitor
**188**	Dipyridamole	BRD-K86301799	94.48	Phosphodiesterase inhibitor
**189**	JNJ-16259685	BRD-K64670467	94.47	Glutamate receptor antagonist
**190**	VU-0415374-1	BRD-K83010055	94.46	Glutamate receptor modulator
**191**	Pidorubicine	BRD-K04548931	94.42	Topoisomerase inhibitor
**192**	KU-C103443N	BRD-A81402010	94.22	CDC inhibitor, Rho associated kinase inhibitor
**193**	Dichlorobenzamil	BRD-K12906962	94.16	Sodium/calcium exchange inhibitor
**194**	Mepacrine	BRD-A45889380	94.14	Cytokine production inhibitor, NFkB pathway inhibitor, TP53 activator
**195**	E-4031	BRD-K41713976	93.96	Potassium channel blocker
**196**	Narciclasine	BRD-K06792661	93.94	Coflilin signaling pathway activator, LIM kinase activator, Rho associated kinase activator
**197**	Mesoridazine	BRD-A14395271	93.80	Dopamine receptor antagonist
**198**	Tranylcypromine	BRD-A43974575	93.79	Monoamine oxidase inhibitor
**199**	Lypressin	BRD-K93331255	93.76	Vasopressin receptor agonist
**200**	Reserpine	BRD-K95921201	93.69	Vesicular monoamine transporter inhibitor
**201**	Abiraterone	BRD-K55301415	93.64	17,20 lyase inhibitor, Androgen biosynthesis inhibitor, Cytochrome P450 inhibitor, Steroid sulfatase inhibitor
**202**	I-OMe-AG-538	BRD-K35377380	93.59	IGF-1 inhibitor
**203**	Somatostatin	BRD-K14681867	93.48	Somatostatin receptor agonist
**204**	Splitomycin	BRD-K27710560	93.37	SIRT inhibitor
**205**	AM-281	BRD-K59419204	93.34	Cannabinoid receptor antagonist
**206**	Sphingosine	BRD-K62959606	93.32	Ceramidase inhibitor
**207**	Hydroxycholesterol	BRD-A36707673	93.30	LXR agonist
**208**	TPCA-1	BRD-K51575138	93.29	IKK inhibitor
**209**	FGIN-1-27	BRD-K09778810	93.26	Inositol monophosphatase inhibitor
**210**	Trichostatin-a	BRD-K68202742	93.06	HDAC inhibitor, CDK activator, ID1 inhibitor
**211**	Hexylresorcinol	BRD-K99946902	92.99	Local anesthetic
**212**	Epicatechin	BRD-K50660797	92.96	Bacterial DNA gyrase inhibitor, Cyclooxygenase inhibitor, DNA polymerase inhibitor
**213**	RS-17053	BRD-K76840893	92.95	Adrenergic receptor antagonist
**214**	NSC-663284	BRD-K03109492	92.78	CDC inhibitor
**215**	L-165041	BRD-K40656405	92.55	PPAR receptor agonist
**216**	ML-7	BRD-K93201660	92.46	Myosin light chain kinase inhibitor
**217**	Alisertib	BRD-K75295174	92.44	Aurora kinase inhibitor
**218**	GR-127935	BRD-K11911061	92.37	Serotonin receptor antagonist
**219**	Clobenpropit	BRD-K71430621	92.36	Histamine receptor antagonist
**220**	NNC-55-0396	BRD-K78122587	92.31	T-type calcium channel blocker
**221**	Barasertib	BRD-K63923597	92.27	Aurora kinase inhibitor
**222**	Benidipine	BRD-A35519318	92.25	Calcium channel blocker
**223**	Sertraline	BRD-K82036761	92.24	Serotonin receptor antagonist
**224**	ZM-447439	BRD-K72703948	92.16	Aurora kinase inhibitor
**225**	BIBX-1382	BRD-K70914287	92.15	EGFR inhibitor, Tyrosine kinase inhibitor
**226**	Immethridine	BRD-K49519092	92.15	Histamine receptor agonist
**227**	MR-16728	BRD-A30590053	92.15	Acetylcholine release enhancer, Acetylcholine release stimulant
**228**	Heraclenol	BRD-A77050075	92.11	Vitamin K antagonist
**229**	NU-7441	BRD-K00337317	92.10	DNA dependent protein kinase inhibitor, P-glycoprotein inhibitor
**230**	Hyoscyamine	BRD-K40530731	91.85	Acetylcholine receptor antagonist
**231**	m-chlorophenylbiguanide	BRD-K36965586	91.76	Serotonin receptor agonist
**232**	Prostaglandin-b2	BRD-K82865713	91.75	cAMP inhibitor
**233**	BML-ST330	BRD-A77118605	91.59	Phospholipase inhibitor
**234**	STO-609	BRD-K52620403	91.57	Calmodulin antagonist
**235**	Tyrphostin-AG-556	BRD-K14441456	91.50	EGFR inhibitor
**236**	Corynanthine	BRD-K06467078	91.46	Adrenergic receptor antagonist
**237**	PD-102807	BRD-A89337244	91.31	Acetylcholine receptor antagonist
**238**	Norgestrel	BRD-A50928468	91.25	Progesterone receptor agonist
**239**	Telmisartan	BRD-K73999723	91.25	Angiotensin receptor antagonist

**240**	BMY-45778	BRD-K84895041	91.22	IP1 prostacyclin receptor agonist
**241**	Dihydrosamidin	BRD-K63945320	91.19	Phospholipase inhibitor, Nitric oxide production inhibitor, platelet activating factor receptor antagonist
**242**	HG-6-64-01	BRD-U37049823	91.09	RAF inhibitor
**243**	KUC104502N	BRD-K24538644	91.06	—
**244**	Formestane	BRD-A31801025	91.00	Aromatase inhibitor
**245**	BRD-K64835161	BRD-K64835161	90.93	—
**246**	M2-PK-activator	BRD-K80672993	90.89	—
**247**	Cetraxate	BRD-K48932581	90.81	Mucus protecting agent
**248**	Terbinafine	BRD-K68132782	90.76	Fungal squalene epoxidase inhibitor
**249**	Phosphodiesterase-V-inhibitor-II	BRD-K68873215	90.69	Phosphodiesterase inhibitor
**250**	Ponalrestat	BRD-K68332390	90.68	Aldose reductase inhibitor
**251**	Phenytoin	BRD-K55930204	90.67	Hydantoin antiepileptic
**252**	Phylloquinone	BRD-A55815733	90.64	Vitamin K, Γ carboxylase enzyme
**253**	AZD-8055	BRD-K69932463	90.64	MTOR inhibitor
**254**	PHA-665752	BRD-K95435023	90.53	c-Met inhibitor
**255**	PD-184352	BRD-K05104363	90.52	MEK inhibitor
**256**	RU-28318	BRD-A92585442	90.43	Cytochrome P450 inhibitor
**257**	Fenoldopam	BRD-A50684349	90.38	Dopamine receptor agonist
**258**	Camptothecin	BRD-A30437061	90.32	Topoisomerase inhibitor
**259**	Tretinoin	BRD-K06926592	90.31	Retinoid receptor agonist, Retinoid receptor ligand
**260**	Metixene	BRD-A33711280	90.16	Acetylcholine receptor antagonist
**261**	Tetrahydropalmatine	BRD-A43940795	90.12	Serotonin release inhibitor
**262**	YM-976	BRD-K12932420	90.09	Phosphodiesterase inhibitor
**263**	Alaproclate	BRD-A14966924	90.03	Serotonin receptor antagonist

Table S3B. 275 compounds with CMap scores < -90, which can potentially elicit anti-cytokine activity against hyperinflammation in SARS-CoV-2-infected A549-ACE2 cells

Index	Compound name	CMap ID	CMap score	Mechanism of Action (MOA)
**1**	n-(3-acetamidophenyl)-3-chlorobenzamide	BRD-K61217870	-100	Glutamate receptor antagonist
**2**	BI-78D3	BRD-K73982490	-99.98	JNK inhibitor
**3**	Xaliproden	BRD-K88358234	-99.98	Serotonin receptor agonist
**4**	Rhamnetin	BRD-K37206356	-99.98	HDAC inhibitor
**5**	MR-16728	BRD-A30590053	-99.98	Acetylcholine release enhancer or stimulant
**6**	Palonosetron	BRD-K08924299	-99.98	Serotonin receptor antagonist
**7**	Clarithromycin	BRD-K49668410	-99.98	Bacterial 50S ribosomal subunit inhibitor
**8**	SAL-1	BRD-K40213712	-99.98	Adenosine receptor antagonist
**9**	Nimodipine	BRD-A58048407	-99.98	Calcium channel blocker
**10**	Isoliquiritigenin	BRD-K33583600	-99.98	Guanylate cyclase activator
**11**	Cyclazosin	BRD-A37837077	-99.98	Adrenergic receptor antagonist
**12**	Eicosatetraynoic-acid	BRD-K06080977	-99.98	Cyclooxygenase inhibitor, Lipoxygenase inhibitor
**13**	Oxantel	BRD-K66019333	-99.96	Anthelmintic
**14**	Nor-binaltorphimine	BRD-A11135865	-99.96	Opioid receptor antagonist
**15**	PCA-4248	BRD-A29289453	-99.95	Platelet activating factor receptor antagonist
**16**	Ketanserin	BRD-K49671696	-99.95	Serotonin receptor antagonist
**17**	Tyrphostin-AG-82	BRD-K03670461	-99.94	EGFR inhibitor
**18**	Azelastine	BRD-A68888262	-99.94	Histamine receptor antagonist
**19**	Diethylstilbestrol	BRD-K45330754	-99.93	Estrogen receptor agonist
**20**	Raltegravir	BRD-K05658747	-99.93	HIV integrase inhibitor
**21**	KI-16425	BRD-A25569250	-99.93	Lysophosphatidic acid receptor antagonist
**22**	Pyroxamide	BRD-K11663430	-99.93	HDAC inhibitor
**23**	Maprotiline	BRD-K03319035	-99.93	Norepinephrine reuptake inhibitor, Tricyclic antidepressant
**24**	Reserpic-acid	BRD-K32755366	-99.91	Norepinephrine transporter inhibitor
**25**	Fostamatinib	BRD-K20285085	-99.91	SYK inhibitor
**26**	Y-27632	BRD-K44084986	-99.91	Rho associated kinase inhibitor
**27**	Dexketoprofen	BRD-K43764301	-99.9	Cyclooxygenase inhibitor
**28**	EMF-bca1-60	BRD-K68437527	-99.9	caspase inhibitor
**29**	Fluphenazine	BRD-K55127134	-99.9	Dopamine receptor antagonist
**30**	Gabazine	BRD-K93280214	-99.89	GABA receptor antagonist
**31**	α-estradiol	BRD-A60070924	-99.88	Estrogen receptor agonist

**32**	Benzydamine	BRD-K76133116	-99.88	Membrane integrity inhibitor, Prostanoid receptor antagonist, Prostanoid receptor inhibitor
**33**	Navitoclax	BRD-K82746043	-99.88	BCL inhibitor
**34**	Nifurtimox	BRD-A00100033	-99.86	DNA inhibitor
**35**	Thenoyltrifluoroacetone	BRD-K00959089	-99.86	Chelating agent
**36**	NVP-AUY922	BRD-K41859756	-99.86	HSP inhibitor
**37**	BRD-K64835161	BRD-K64835161	-99.85	NA
**38**	Atorvastatin	BRD-U88459701	-99.85	HMGCR (HMG CoA reductase) inhibitor
**39**	Securinine	BRD-A25775766	-99.82	GABA receptor antagonist, TP53 activator
**40**	Nikkomycin	BRD-A74771556	-99.82	Chitin inhibitor
**41**	Zuclopenthixol	BRD-K28761384	-99.78	Dopamine receptor antagonist
**42**	Temozolomide	BRD-K32107296	-99.78	DNA alkylating agent
**43**	HY-11007	BRD-K97056771	-99.77	BCR-ABL kinase inhibitor
**44**	Salsolinol	BRD-K99595596	-99.74	Monoamine oxidase inhibitor, Tyrosine hydroxylase inhibitor
**45**	SCH-28080	BRD-K55748775	-99.73	ATPase inhibitor
**46**	Retinol	BRD-K13927029	-99.72	Retinoid receptor ligand
**47**	SB-216763	BRD-K59184148	-99.72	Glycogen synthase kinase inhibitor
**48**	YS-035	BRD-K06208435	-99.71	Calcium channel blocker
**49**	Bisoprolol	BRD-A89175223	-99.69	Adrenergic receptor antagonist
**50**	Carteolol	BRD-A42167015	-99.62	Adrenergic receptor antagonist
**51**	TER-14687	BRD-A33833419	-99.62	Inhibitor of translocation of PKCq in T cells
**52**	Selegiline	BRD-K86434416	-99.59	Monoamine oxidase inhibitor
**53**	Triptolide	BRD-A13122391	-99.58	RNA polymerase inhibitor
**54**	Lisuride	BRD-K88871508	-99.57	Dopamine receptor agonist
**55**	Topiramate	BRD-K29653726	-99.57	Carbonic anhydrase inhibitor, Glutamate receptor antagonist, Kainate receptor antagonist
**56**	Berbamine	BRD-K50464341	-99.55	Calmodulin antagonist
**57**	Hexamethyleneamiloride	BRD-K40990712	-99.53	Sodium/hydrogen antiport inhibitor
**58**	MW-STK33-3B	BRD-K64310881	-99.52	Potassium channel activator
**59**	MLN-4924	BRD-K67844266	-99.5	Nedd activating enzyme inhibitor
**60**	EHNA	BRD-K27450477	-99.48	Adenosine deaminase inhibitor
**61**	Chlorprothixene	BRD-K59058766	-99.46	Dopamine receptor antagonist
**62**	DUP-697	BRD-K06221026	-99.45	Cyclooxygenase inhibitor
**63**	Chlorphenamine	BRD-A04553218	-99.45	Histamine receptor antagonist
**64**	NAS-181	BRD-A23683907	-99.38	Serotonin receptor antagonist
**65**	Linsitinib	BRD-K08589866	-99.37	IGF-1 inhibitor, insulin inhibitor, ARF6 and TBK1 activator
**66**	YC-1	BRD-K60476892	-99.34	Guanylyl cyclase activator
**67**	Olanzapine	BRD-K18895904	-99.32	Dopamine receptor/serotonin receptor antagonist
**68**	Orantinib	BRD-K91696562	-99.3	FGFR, VEGFR, PDGFR inhibitor
**69**	Phenelzine	BRD-K87024524	-99.3	Monoamine oxidase inhibitor
**70**	TGX-221	BRD-A41692738	-99.29	PI3K inhibitor
**71**	Latrepirdine	BRD-K55703048	-99.29	Glutamate receptor antagonist
**72**	PU-H71	BRD-K36529613	-99.28	HSP inhibitor
**73**	Bromocriptine	BRD-A69960130	-99.24	Dopamine receptor agonist
**74**	Syrosingopine	BRD-K14200658	-99.21	Vesicular monoamine transporter inhibitor
**75**	UNC-0321	BRD-K74236984	-99.21	Histone lysine methyltransferase inhibitor
**76**	BRD-A80383043	BRD-A80383043	-99.19	Glutamate receptor agonist and/or antagonist
**77**	Trifluoperazine	BRD-K89732114	-99.17	Dopamine receptor antagonist
**78**	AQ-RA741	BRD-K81729199	-99.16	Acetylcholine receptor antagonist
**79**	Bromfenac	BRD-K47679368	-99.14	Cyclooxygenase inhibitor
**80**	Oxaprozin	BRD-K25394294	-99.14	Cyclooxygenase inhibitor
**81**	CGS-20625	BRD-K68103045	-99.12	Benzodiazepine receptor agonist, GABA benzodiazepine site receptor partial agonist
**82**	Rucaparib	BRD-K88560311	-99.11	PARP inhibitor
**83**	o-3M3FBS	BRD-K46384212	-99.09	phospholipase activator
**84**	L-655240	BRD-K89402695	-99.07	Thromboxane receptor antagonist
**85**	L-750667	BRD-K28806945	-99.04	Dopamine receptor antagonist
**86**	Daunorubicin	BRD-K43389675	-98.99	RNA synthesis inhibitor, Topoisomerase inhibitor
**87**	Profenamine	BRD-A16311756	-98.98	Butyrylcholinesterase inhibitor, Cholinergic receptor antagonist
**88**	Saracatinib	BRD-K19540840	-98.97	SRC inhibitor
**89**	Trazodone	BRD-K70778732	-98.96	Adrenergic receptor antagonist, Serotonin receptor antagonist, Serotonin reuptake inhibitor
**90**	Valproic-acid	BRD-K41260949	-98.93	HDAC inhibitor
**91**	Medetomidine	BRD-A66563878	-98.93	Adrenergic receptor agonist
**92**	Piperine	BRD-K59522102	-98.92	Monoamine oxidase inhibitor

**93**	Pyrazinamide	BRD-K28667793	-98.83	Fatty acid synthase inhibitor
**94**	Fraxidin	BRD-K66944906	-98.83	Carbonic anhydrase inhibitor
**95**	Larixinic-acid	BRD-K40619305	-98.78	Compound that interacts with metal centers
**96**	Midodrine	BRD-A79981887	-98.74	Adrenergic receptor agonist
**97**	XAV-939	BRD-K12762134	-98.73	Tankyrase inhibitor
**98**	AICA-ribonucleotide	BRD-A67373739	-98.7	AMPK activator
**99**	PIK-90	BRD-K99818283	-98.66	PI3K inhibitor
**100**	PNU-22394	BRD-K16551401	-98.63	Serotonin receptor agonist
**101**	AY-9944	BRD-K03642198	-98.62	Hedgehog pathway modulator
**102**	Gavestinel	BRD-K49890030	-98.59	Glutamate receptor antagonist
**103**	Foliosidine	BRD-A49734948	-98.54	Plant alkaloid
**104**	Naftopidil	BRD-A01787639	-98.48	Adrenergic receptor antagonist
**105**	GDC-0941	BRD-K52911425	-98.38	PI3K inhibitor
**106**	Clonidine	BRD-K98530306	-98.37	Adrenergic receptor agonist
**107**	CGP-54626	BRD-A55369275	-98.35	GABA receptor antagonist
**108**	Tosyllysyl-chloromethyl-ketone	BRD-K10136726	-98.32	Chymotrypsin inhibitor
**109**	Indatraline	BRD-K01649396	-98.31	Norepinephrine transporter inhibitor
**110**	9-methyl-5H-6-thia-4,5-diaza-chrysene-6,6-dioxide	BRD-K14696368	-98.28	NFkB pathway inhibitor
**111**	NNC-05-2090	BRD-K85015012	-98.27	GAT inhibitor, GABA uptake inhibitor
**112**	3-matida	BRD-A87125127	-98.19	Glutamate receptor antagonist
**113**	Phenothiazine	BRD-K59597909	-98.17	Dopamine receptor antagonist
**114**	Piribedil	BRD-K47936004	-98.16	Dopamine receptor agonist
**115**	Mebeverine	BRD-A09467419	-98.09	Acetylcholine receptor antagonist
**116**	GANT-58	BRD-K64451768	-98.06	GLI antagonist
**117**	RITA	BRD-K00317371	-98.03	MDM inhibitor
**118**	Pirenperone	BRD-K25224017	-98.01	Serotonin receptor antagonist
**119**	KIN001-244	BRD-K09186807	-98.01	Phosphoinositide dependent kinase inhibitor
**120**	Ozagrel	BRD-K19525698	-97.97	Thromboxane synthase inhibitor
**121**	PP-30	BRD-K30677119	-97.96	RAF inhibitor
**122**	CNQX	BRD-K53545112	-97.96	Glutamate receptor antagonist
**123**	STO-609	BRD-K52620403	-97.91	Calmodulin antagonist
**124**	Loperamide	BRD-K61250553	-97.74	Opioid receptor agonist
**125**	Dichloroacetic-acid	BRD-K13664374	-97.69	Pyruvate dehydrogenase kinase inhibitor
**126**	PI-103	BRD-K67868012	-97.66	MTOR inhibitor, PI3K inhibitor
**127**	Metoclopramide	BRD-K75641298	-97.54	Dopamine receptor and serotonin receptor antagonist
**128**	Spironolactone	BRD-K90027355	-97.54	Mineralocorticoid receptor antagonist
**129**	Loratadine	BRD-K82795137	-97.51	Histamine receptor antagonist
**130**	Pirfenidone	BRD-K96862998	-97.45	TGF β receptor inhibitor
**131**	ICI-89406	BRD-A03359064	-97.34	Adrenergic receptor antagonist
**132**	Clebopride	BRD-K17294426	-97.31	Dopamine receptor antagonist
**133**	Prostaglandin-a1	BRD-K04010869	-97.3	HSP inducer, NFkB pathway inhibitor
**134**	Butylparaben	BRD-K08287586	-97.27	DNA synthesis inhibitor
**135**	Testosterone	BRD-A48720949	-97.13	androgen receptor agonist
**136**	WZ-4002	BRD-K72420232	-97.11	EGFR inhibitor
**137**	PLX-4720	BRD-K16478699	-97.01	RAF inhibitor
**138**	Darinaparsin	BRD-K35723520	-97.01	Apoptosis stimulant
**139**	PTB1	BRD-K16554956	-96.94	AMPK activator
**140**	Alosetron	BRD-K46742498	-96.93	Serotonin receptor antagonist
**141**	U-99194	BRD-K70281171	-96.87	Dopamine receptor antagonist
**142**	Otenzepad	BRD-A00520476	-96.85	Acetylcholine receptor antagonist
**143**	Fursultiamine	BRD-A71157293	-96.84	Vitamin B
**144**	Piperacetazine	BRD-K16277217	-96.79	Dopamine receptor antagonist
**145**	SD-169	BRD-K91904471	-96.77	p38 MAPK inhibitor
**146**	Liothyronine	BRD-K89152108	-96.77	Thyroid hormone stimulant
**147**	Mepacrine	BRD-A45889380	-96.77	Cytokine production inhibitor, NFkB pathway inhibitor, TP53 activator
**148**	Nicotine	BRD-K05395900	-96.71	Acetylcholine receptor agonist
**149**	TG-101348	BRD-K12502280	-96.66	FLT3 inhibitor, JAK inhibitor
**150**	Quinpirole	BRD-A85280935	-96.54	Dopamine receptor agonist
**151**	Mafenide	BRD-K30649484	-96.53	Carbonic anhydrase inhibitor
**152**	Dephostatin	BRD-K60274257	-96.5	Tyrosine phosphatase inhibitor
**153**	Cisapride	BRD-K06895174	-96.49	Serotonin receptor agonist
**154**	Dicyclohexylurea	BRD-K81521265	-96.48	Epoxide hydrolase inhibitor
**155**	m-chlorophenylbiguanide	BRD-K36965586	-96.47	Serotonin receptor agonist

**156**	Auraptene	BRD-K85013741	-96.45	Nitric oxide production inhibitor
**157**	Alprenolol	BRD-A00993607	-96.44	Adrenergic receptor antagonist
**158**	TPCA-1	BRD-K51575138	-96.39	IKK inhibitor
**159**	Sertraline	BRD-K82036761	-96.37	Serotonin receptor antagonist
**160**	AC-55649	BRD-K93176058	-96.37	Retinoid receptor agonist
**161**	CDK1-5-inhibitor	BRD-K87932577	-96.35	CDK inhibitor, Glycogen synthase kinase inhibitor
**162**	D-64406	BRD-K27665173	-96.25	PDGFR receptor inhibitor
**163**	Fipronil	BRD-A50675702	-96.24	GABA gated chloride channel blocker
**164**	RO-25-6981	BRD-K51541829	-96.14	Ionotropic glutamate receptor antagonist, Monamine transporter modulator
**165**	BIIB021	BRD-K51967704	-96.11	HSP inhibitor
**166**	AZD-6482	BRD-K58772419	-96.1	PI3K inhibitor
**167**	EMD-386088	BRD-K47659338	-96.09	Serotonin receptor agonist
**168**	CITCO	BRD-K53263234	-96.07	CAR agonist
**169**	Exemestane	BRD-A73741725	-95.97	Aromatase inhibitor
**170**	GR-206	BRD-K00184207	-95.95	Aryl hydrocarbon receptor ligand
**171**	Dasatinib	BRD-K49328571	-95.91	BCR-ABL kinase inhibitor, Ephrin inhibitor, KIT inhibitor, PDGFR receptor inhibitor, SRC inhibitor, Tyrosine kinase inhibitor
**172**	MDM2-inhibitor	BRD-K84987553	-95.89	MDM inhibitor
**173**	Aminomethyltransferase	BRD-A28318179	-95.8	Nitric oxide synthase inhibitor
**174**	BRL-52537	BRD-A37347161	-95.7	Opioid receptor agonist
**175**	Amoxapine	BRD-K02265150	-95.68	Norepinephrine reuptake inhibitor
**176**	RO-08-2750	BRD-K00486786	-95.68	NGF binding inhibitor
**177**	Flutamide	BRD-K28307902	-95.68	Androgen receptor antagonist
**178**	DMBI	BRD-K96084870	-95.63	PDGFR receptor inhibitor, VEGFR inhibitor
**179**	Carmoxirole	BRD-K82484965	-95.61	Dopamine receptor agonist
**180**	Taurodeoxycholic-acid	BRD-K33572481	-95.59	Bile acid
**181**	Bupropion	BRD-A05186015	-95.57	Dopamine uptake inhibitor
**182**	Chlordiazepoxide	BRD-K86595100	-95.49	Benzodiazepine receptor agonist
**183**	Roscovitine	BRD-K07691486	-95.37	CDK inhibitor
**184**	ALW-II-38-3	BRD-K68191783	-95.33	Ephrin inhibitor
**185**	Ornidazole	BRD-A42759514	-95.22	Antiprotozoal
**186**	Iodophenpropit	BRD-K51918615	-95.18	Histamine receptor antagonist
**187**	Prima-1-met	BRD-K49456190	-95.18	thioredoxin inhibitor
**188**	EI-247	BRD-K32710582	-95.17	IGF-1 inhibitor
**189**	MK-2206	BRD-K68065987	-95.16	AKT inhibitor
**190**	BMS-299897	BRD-K02950022	-95.14	γ secretase inhibitor
**191**	Promazine	BRD-K06980535	-95.11	Dopamine receptor antagonist
**192**	CGP-60474	BRD-K79090631	-95.09	CDK inhibitor
**193**	PF-04217903	BRD-K73319509	-95.06	c-Met inhibitor
**194**	Pantoprazole	BRD-A22380646	-95.02	ATPase inhibitor
**195**	Norgestimate	BRD-A04756508	-94.95	Progesterone receptor agonist
**196**	Mead-ethanolamide	BRD-K09764130	-94.82	Cannabinoid receptor agonist
**197**	CL-82198	BRD-K00675675	-94.73	Metalloproteinase inhibitor
**198**	HLI-373	BRD-K17349619	-94.73	MDM inhibitor
**199**	Nifedipine	BRD-K96354014	-94.71	Calcium channel blocker
**200**	Sildenafil	BRD-K50128260	-94.71	Phosphodiesterase inhibitor
**201**	ICI-199441	BRD-K73290745	-94.63	Opioid receptor agonist
**202**	Ipsapirone	BRD-K90574421	-94.61	Serotonin receptor agonist
**203**	Milrinone	BRD-K67080878	-94.49	Phosphodiesterase inhibitor
**204**	Cotinine	BRD-K94144010	-94.35	Nicotine metabolite
**205**	Etilefrine	BRD-A09925278	-94.23	Adrenergic receptor agonist
**206**	Thioproperazine	BRD-K08619574	-94.19	Dopamine receptor antagonist
**207**	Acadesine	BRD-A95696820	-94.15	AMPK activator
**208**	Danazol	BRD-A92537424	-94.09	Estrogen receptor antagonist, Progesterone receptor agonist
**209**	z-prolyl-prolinal	BRD-K60174629	-94.04	Prolyl endopeptidase inhibitor
**210**	Pravastatin	BRD-K60511616	-94.03	HMGCR inhibitor
**211**	PP-2	BRD-K95785537	-94.01	SRC inhibitor
**212**	BRD-K63784565	BRD-K63784565	-94.01	Topoisomerase inhibitor
**213**	Geldanamycin	BRD-A19500257	-93.9	HSP inhibitor
**214**	Verapamil	BRD-A09533288	-93.88	Calcium channel blocker
**215**	Amylocaine	BRD-A09062839	-93.70	Local anesthetic
**216**	Anagrelide	BRD-K62200014	-93.38	Phosphodiesterase inhibitor
**217**	JAK3-Inhibitor-II	BRD-K52850071	-93.37	JAK inhibitor
**218**	Felbamate	BRD-K99107520	-93.35	Glutamate receptor antagonist

**219**	BP-554	BRD-K45479396	-93.33	Serotonin receptor agonist
**220**	Dicycloverine	BRD-K68507560	-93.3	Acetylcholine receptor antagonist
**221**	Nicorandil	BRD-K97752965	-93.29	Nitric oxide donor, Potassium channel activator
**222**	SCH-442416	BRD-K46469693	-93.27	Adenosine receptor antagonist
**223**	Carpindolol	BRD-A15530910	-93.19	Adrenergic receptor antagonist, serotonin receptor antagonist
**224**	VU-0420363-1	BRD-K59633790	-92.96	SARS coronavirus 3C-like protease inhibitor
**225**	Oxybutynin	BRD-A65013509	-92.9	Acetylcholine receptor antagonist
**226**	SA-792541	BRD-K68143200	-92.8	CDC inhibitor
**227**	Dipropyl-5ct	BRD-K32645441	-92.74	Serotonin receptor agonist
**228**	Ticlopidine	BRD-K00603606	-92.72	Purinergic receptor antagonist
**229**	SDZ-WAG-994	BRD-A31007383	-92.71	Adenosine receptor agonist
**230**	Meprylcaine	BRD-K65417056	-92.69	Local anesthetic
**231**	Cycloserine	BRD-K87226815	-92.58	Bacterial cell wall synthesis inhibitor
**232**	KIN001-127	BRD-A29901043	-92.54	ITK inhibitor
**233**	Enrofloxacin	BRD-K76534306	-92.54	Bacterial DNA gyrase inhibitor
**234**	Alverine	BRD-K89055274	-92.52	Muscle relaxant
**235**	Bepridil	BRD-A91008255	-92.5	Calcium channel or L-type Ca^{++} channel blocker
**236**	Nefazodone	BRD-K90789829	-92.39	Adrenergic inhibitor, Norepinephrine reuptake inhibitor, Serotonin receptor antagonist, Serotonin reuptake inhibitor
**237**	PSB-11	BRD-K10177585	-92.33	Adenosine receptor antagonist
**238**	Acetyl-geranyl-cysteine	BRD-U01690642	-92.32	Isoprenylated protein methylation inhibitor
**239**	Estradiol	BRD-A18917088	-92.31	Contraceptive agent, Estrogen receptor agonist
**240**	Secoisolariciresinol	BRD-K91733562	-92.2	Antioxidant
**241**	Prostaglandin	BRD-K09436313	-92.13	Prostanoid receptor antagonist
**242**	Alfuzosin	BRD-A09056319	-92.11	Adrenergic receptor antagonist
**243**	Oxybenzone	BRD-K59037100	-92.1	Lipase inhibitor
**244**	KIN001-220	BRD-K53561341	-91.98	Aurora kinase inhibitor
**245**	AZD-8055	BRD-K69932463	-91.85	MTOR inhibitor
**246**	Toltrazuril	BRD-K64514229	-91.83	Antiprotozoal
**247**	Mepyramine	BRD-K97564742	-91.79	Histamine receptor antagonist
**248**	Edaravone	BRD-K35458079	-91.74	Nootropic agent
**249**	FIT	BRD-K17896185	-91.6	Opioid receptor agonist
**250**	Dopamine	BRD-K43887077	-91.51	Dopamine receptor agonist
**251**	Tolterodine	BRD-K54316499	-91.49	Acetylcholine receptor antagonist
**252**	L-BSO	BRD-A47706533	-91.44	Glutathione transferase inhibitor
**253**	Dinoprostone	BRD-K26521938	-91.34	Prostanoid receptor agonist
**254**	GR-144053	BRD-K12120659	-91.31	Integrin antagonist
**255**	O-2050	BRD-K02590140	-91.29	Cannabinoid receptor antagonist
**256**	bis-tyrphostin	BRD-K32906660	-91.25	EGFR inhibitor
**257**	ITE	BRD-K60298136	-91.16	Aryl hydrocarbon receptor agonist
**258**	Nevirapine	BRD-K15502390	-91.15	Reverse transcriptase inhibitor
**259**	GR-235	BRD-K26674531	-91.08	Estrogen receptor agonist, FXR antagonist, Progesterone receptor agonist
**260**	Latrunculin-b	BRD-A19248578	-91.08	Actin polymerization inhibitor, Unidentified pharmacological activity
**261**	AR-C133057XX	BRD-K40892394	-91.08	Nitric oxide synthase inhibitor
**262**	Temefos	BRD-K51805276	-90.87	Cholinesterase inhibitor
**263**	Ilomastat	BRD-K51662849	-90.81	Matrix metalloprotease inhibitor
**264**	SID-26681509	BRD-K08417745	-90.75	Cathepsin inhibitor
**265**	Formestane	BRD-A31801025	-90.66	Aromatase inhibitor
**266**	Iproniazid	BRD-K88568253	-90.6	Monoamine oxidase inhibitor
**267**	Buphenine	BRD-A36267905	-90.46	Adrenergic receptor agonist
**268**	Desipramine	BRD-K60762818	-90.43	Tricyclic antidepressant
**269**	Tyrphostin-46	BRD-K60184833	-90.39	Tyrosine kinase inhibitor
**270**	RS-67506	BRD-K50018155	-90.29	Serotonin receptor partial agonist
**271**	BRD-K34437622	BRD-K34437622	-90.15	Thymidylate synthase inhibitor
**272**	Rotenonic-acid	BRD-K34330170	-90.11	Retinoid receptor antagonist
**273**	PIK-75	BRD-M16762496	-90.1	DNA protein kinase inhibitor, PI3K inhibitor
**274**	Zacopride	BRD-A65615053	-90.01	Serotonin receptor antagonist
**275**	Etifenin	BRD-K63979671	-90.00	Compound used in hepatobiliary scans of the liver

Table S4. Composition of four modules mediating host cell response during SARS-CoV2 infection

Module (# of proteins)	Dominant pathways/processes	Host cell proteins (gene names) in the module
**Viral entry (27 proteins)**	Endocytosis, lysosome pathway	Scavenger receptor class B member 1 (SCARB1); V-type proton ATPase subunit S1 (ATP6AP1); AP-3 complex subunit β-1 (AP3B1); NPC intracellular cholesterol transporter 2 (NPC2); integrin β-1 (ITGB1); Ras-related protein Rab-8A (RAB8A); AP-2 complex subunit α-2 (AP2A2); 1-phosphatidylinositol 3-phosphate 5-kinase (PIKFYVE); transforming protein RhoA (RHOA); Ras-related protein Rab-10 (RAB10); angiotensin-converting enzyme 2 (ACE2); AP-2 complex subunit mu (AP2M1); V-type proton ATPase catalytic subunit A (ATP6V1A); E3 ubiquitin-protein ligase NRDP1 (RNF41); charged multivesicular body protein 2a (CHMP2A); cathepsin B (CTSB); WASH complex subunit 4 (WASHC4); TMPRSS2; Ras-related protein Rab-7a (RAB7A); α-galactosidase A (GLA); spartin (SPART); cathepsin L1 (CTSL); palmitoyl-protein thioesterase 1 (PPT1); ADP-ribosylation factor 6 (ARF6); Ras-related protein Rab-5C (RAB5C); sialidase-1 (NEU1); two pore Ca^{2+} channel protein 2 (TPC2)
**Viral replication and translation (45 proteins)**	DNA replication, RNA transport, RNA degradation, protein processing in ER protein export	Nuclear pore glycoprotein p62 (NUP62); endoplasmic reticulum lectin 1 (ERLEC1); nuclear pore complex protein Nup214 (NUP214); eukaryotic translation initiation factor 4E type 2 (EIF4E2); 60S ribosomal protein L36 (RPL36); vesicular integral-membrane protein VIP36 (LMAN2); exosome complex component RRP40 (EXOSC3); nucleoporin p54 (NUP54); wolframin (WFS1); DNA primase large subunit (PRIM2); signal recognition particle subunit SRP72 (SRP72); nucleotide exchange factor SIL1 (SIL1); regulator of nonsense transcripts 1 (UPF1); selenoprotein S (SELENOS); DNA polymerase α catalytic subunit (POLA1); nuclear pore complex protein Nup88 (NUP88); protein OS-9 (OS9); hypoxia up-regulated protein 1 (HYOU1); mRNA export factor (RAE1); E3 ubiquitin-protein ligase RBX1 (RBX1); exosome complex component RRP4 (EXOSC2); mitochondrial small ribosomal subunit protein uS2m (MRPS2); nuclear pore complex protein Nup98-Nup96 (NUP98); 26S proteasome non-ATPase regulatory subunit 8 (PSMD8); peptide-N (4)- (N-acetyl-β-glucosaminyl)asparagine amidase (NGLY1); nucleoporin p58 (NUP58); ERO1-like protein β (ERO1B); ER degradation-enhancing α -mannosidase-like protein 3 (EDEM3); mitochondrial small ribosomal subunit protein uS5m (MRPS5); DNA primase small subunit (PRIM1); nuclear pore membrane glycoprotein 210 (NUP210); elongin-C (ELOC); signal recognition particle 54 kDa protein (SRP54); elongin-B (ELOB); UDP-glucose:glycoprotein glucosyltransferase 2 (UGGT2); exosome complex component RRP46 (EXOSC5); inosine-5'-monophosphate dehydrogenase 2 (IMPDH2); polyadenylate-binding protein 4 (PABPC4); exosome complex component RRP43 (EXOSC8); DNA polymerase α subunit B (POLA2); signal recognition particle 19 kDa protein (SRP19); pre-mRNA-splicing factor SLU7 (SLU7); cullin-2 (CUL2); mannosyl-oligosaccharide glucosidase (MOGS); polyadenylate-binding protein 1 (PABPC
**Regulation and signaling (27 proteins)**	Ras signaling, autophagy, AMPK signaling, mTOR signaling, PI3K-AKT signaling and insulin signaling	Interleukin-6 receptor subunit α (IL6R); PIK3CA; Ras-related protein Rab-8A (RAB8A); Ras-related protein Ral-A (RALA); mTOR; serine/threonine-protein kinase TBK1 (TBK1); AKT1; NFkB1; guanine nucleotide-binding protein G (I)/G (S)/G (O) subunit γ-5 (GNG5); eukaryotic translation initiation factor 4E type 2 (EIF4E2); cAMP-dependent protein kinase type II-α regulatory subunit (PRKAR2A); Ras-related protein Rab-2A (RAB2A); MYD88; V-type proton ATPase catalytic subunit A (ATP6V1A); collagen α -1 (VI) chain (COL6A1); Ras-related protein Rab-14 (RAB14); MAPK1; RHOA; RAB10; cAMP-dependent protein kinase type II-β regulatory subunit (PRKAR2B); integrin β -1 (ITGB1); guanine nucleotide-binding protein G (I)/G (S)/G (T) subunit β-1 (GNB1); ARF6; RAB5C; evolutionarily conserved signaling intermediate in Toll pathway (ECSIT); cAMP-dependent protein kinase catalytic subunit α (PRKACA); NFATC1

**Immune response (32 proteins)**	Interferon-, TLR-, chemokine-, NFkB-, RIG-like receptor-, B-cell receptor-, T cell receptor- and HIF-1-signaling	Myeloid differentiation primary response protein MyD88 (MYD88); MAPK1; STAT3; cullin-2 (CUL2); heme oxygenase 1 (HMOX1); ELOB; receptor-interacting serine/threonine-protein kinase 1 (RIPK1); interleukin-17 receptor A (IL17RA); casein kinase II subunit β (CSNK2B); serine/threonine-protein kinase mTOR (MTOR); inhibin β E chain (INHBE); cAMP-dependent protein kinase catalytic subunit α (PRKACA); guanine nucleotide-binding protein G (I)/G (S)/G (T) subunit β-1 (GNB1); NLR family member X1 (NLRX1); ELKS/Rab6-interacting/CAST family member 1 (ERC1); RHOA; growth/differentiation factor 15 (GDF15); serine/threonine-protein kinase TBK1 (TBK1); IL6R; AKT1; casein kinase II subunit α (CSNK2A2); guanine nucleotide-binding protein G (I)/G (S)/G (O) subunit γ-5 (GNG5); NFATC1; TANK-binding kinase 1-binding protein 1 (TBKBP1); PIK3CA; cathepsin B (CTSB); E3 ubiquitin-protein ligase RBX1 (RBX1); NFkB1; ELOC; eukaryotic translation initiation factor 4E type 2 (EIF4E2); tissue-type plasminogen activator (PLAT); ARF6

Table S5. **Top-ranking 64 compounds involved in four disease modules, rank-ordered by the proximity of the corresponding targets to the disease modules (*)**

Viral entry			Viral replication & translation		
**Index**	**Compound name**	**z-score**	**Index**	**Compound name**	**z-score**
**1**	SN-38	-2.97E+00	**1**	Methimazole	-3.93E+00
**2**	Hexylresorcinol	-1.87E+00	**2**	Mefenamic acid	-2.62E+00
**3**	GSK-1904529A	-1.79E+00	**3**	Fludarabine	-2.50E+00
**4**	Linsitinib	-1.79E+00	**4**	TGX-221	-1.87E+00
**5**	Sphingosine	-1.48E+00	**5**	Ibuprofen	-1.50E+00
**6**	Semaxanib	-1.09E+00	**6**	Razoxane	-1.38E+00
**7**	Azathioprine	-1.08E+00	**7**	Somatostatin	-8.50E-01
**8**	Imipramine	-8.18E-01	**8**	NU-7441	-8.39E-01
**9**	KU-55933	-7.96E-01	**9**	AS-605240	-7.83E-01
**10**	Mesoridazine	-7.43E-01	**10**	Leflunomide	-7.80E-01
**11**	Alisertib	-7.13E-01	**11**	NU-7026 (LY-293646)	-6.66E-01
**12**	Salmeterol	-6.89E-01	**12**	Aspirin	-6.62E-01
**13**	Terbutaline	-6.89E-01	**13**	Tolazoline	-4.83E-01
**14**	NBI-27914	-6.56E-01	**14**	Clobenpropit	-4.83E-01
**15**	Desoxycorticosterone	-6.45E-01	**15**	JNJ-16259685	-4.79E-01
**16**	GR-127935	-6.45E-01	**16**	VU-0415374-1	-4.79E-01
**17**	Terfenadine	-4.98E-01	**17**	NBI-27914	-4.79E-01
**18**	Dactolisib	-4.57E-01	**18**	Ponalrestat	-4.72E-01
**19**	JNJ-16259685	-4.48E-01	**19**	Brompheniramine	-2.93E-01
**20**	VU-0415374-1	-4.48E-01	**20**	Oxybutynin	-2.44E-01
**21**	SCH-23390	-4.46E-01	**21**	Ipratropium	-2.44E-01
**22**	Ezetimibe	-4.42E-01	**22**	Procyclidine	-2.44E-01
**23**	Brompheniramine	-4.39E-01	**23**	Hyoscyamine	-2.44E-01
**24**	Desipramine	-4.03E-01	**24**	Metixene	-2.44E-01
**25**	Oxybutynin	-3.61E-01	**25**	Rescinnamine	-2.28E-01

Cell signaling & regulation			Immune response		
**Index**	**Compound name**	**z-score**	**Index**	**Compound name**	**z-score**
**1**	PKCβ-inhibitor	-4.10E+00	**1**	Fostamatinib	-5.16E+00
**2**	Dactolisib	-3.91E+00	**2**	NVP-TAE684	-4.47E+00
**3**	Fostamatinib	-3.48E+00	**3**	PKCβ-inhibitor	-4.37E+00
**4**	NVP-TAE684	-3.24E+00	**4**	Bosutinib	-3.59E+00
**5**	Wortmannin	-3.19E+00	**5**	Wortmannin	-3.29E+00
**6**	PDE-V-Inhibitor II	-2.83E+00	**6**	**WHI-P154 (JAK3-Inhibitor-II)**	-3.04E+00
**7**	TPCA-1	-2.56E+00	**7**	TPCA-1	-3.04E+00
**8**	STO-609	-2.50E+00	**8**	Dactolisib	-2.88E+00
**9**	Bosutinib	-2.39E+00	**9**	NU-7441	-2.77E+00
**10**	Benidipine	-2.37E+00	**10**	PDE-V-Inhibitor II	-2.68E+00
**11**	Dipyridamole	-2.22E+00	**11**	Benidipine	-2.32E+00
**12**	SCH-23390	-2.00E+00	**12**	Dipyridamole	-2.06E+00
**13**	PI-103	-1.92E+00	**13**	Semaxanib	-2.06E+00
**14**	**WHI-P154 (JAK3-Inhibitor-II)**	-1.88E+00	**14**	AS-605240	-1.89E+00
**15**	AS-605240	-1.88E+00	**15**	KU-0063794	-1.87E+00
**16**	NU-7441	-1.81E+00	**16**	Temsirolimus	-1.87E+00
**17**	Alisertib	-1.80E+00	**17**	**AZD-8055**	-1.87E+00

(*)

**18**	KU-0063794	-1.79E+00	**18**	STO-609	-1.84E+00
**19**	Temsirolimus	-1.79E+00	**19**	PI-103	-1.81E+00
**20**	**AZD-8055**	-1.79E+00	**20**	Pimozide	-1.79E+00
**21**	Torin-1	-1.72E+00	**21**	SCH-23390	-1.70E+00
**22**	Semaxanib	-1.66E+00	**22**	Torin-1	-1.69E+00
**23**	Reserpine	-1.54E+00	**23**	Reserpine	-1.65E+00
**24**	Telmisartan	-1.48E+00	**24**	Leflunomide	-1.58E+00
**25**	Terfenadine	-1.36E+00	**25**	Aspirin	-1.40E+00

highlighted compounds/drugs have been experimentally tested. z-score is a measure of proximity, the lower scores indicating closer proximity. 25 compounds with the lowest proximity score are listed for each module. Several compounds participate in multiple modules resulting in 64 distinct compounds in the listed four modules.

Table S6A Grouping of 64 potentially antiviral compounds/drugs into clusters based on their interaction patterns with their targets (*)

Cluster index	Index	Compound name	PubChem ID	DrugBank ID
1	1	Ipratropium	657309	DB00332
	2	Terfenadine	5405	DB00342
	3	Brompheniramine	6834	DB00835
	4	Metixene	4167	DB00340
	5	Oxybutynin	4634	DB01062
	6	Procyclidine	4919	DB00387
	7	Hyoscyamine	154417	DB00424
2	8	Desipramine	2995	DB01151
	9	Imipramine	3696	DB00458
	10	Mesoridazine	4078	DB00933
	11	SCH-23390	5018	NA
	12	Pimozide	16362	DB01100
	13	Desoxycorticosterone	6166	NA
	14	GR-127935	107780	NA
3	15	AZD-8055	25262965	DB12774
	16	KU-0063794	16736978	NA
	17	Temsirolimus	6918289	DB06287
4	18	Wortmannin	312145	DB08059
	19	Torin-1	49836027	NA
	20	Dactolisib	11977753	NA
	21	PI-103	9884685	NA
	22	AS-605240	5289247	DB04769
	23	NU-7441	11327430	NA
	24	TGX-221	9907093	NA
5	25	GSK-1904529A	25124816	NA
	26	Linsitinib	11640390	NA
6	27	Salmeterol	5152	DB00938
	28	Terbutaline	5403	DB00871
7	29	STO-609	3467590	NA
	30	Bosutinib	5328940	DB06616
	31	Fostamatinib	11671467	DB12010
	32	NVP-TAE684	16038120	NA
	33	PKCβ-inhibitor	6419755	NA
	34	WHI-P154 (JAK3-Inhibitor-II)	3795	NA
	35	Semaxanib	5329098	NA
	36	TPCA-1	9903786	NA
8	37	Tolazoline	5504	DB00797
	38	Clobenpropit	2790	NA
9	39	NU-7026 (LY-293646)	9860529	NA
10	40	SN-38	104842	DB05482
	41	Hexylresorcinol	3610	DB11254
11	42	Ponalrestat	5278	NA
	43	Aspirin	2244	DB00945
	44	Mefenamic acid	4044	DB00784
	45	Ibuprofen	3672	DB01050
12	46	Alisertib	24771867	DB05220
13	47	JNJ-16259685	11313361	NA
	48	VU-0415374-1	46869940	NA
14	49	PDE-V-Inhibitor II	9844109	NA
	50	Dipyridamole	3108	DB00975
15	51	Telmisartan	65999	DB00966
16	52	Leflunomide	3899	DB01097

**17**	53	Methimazole	1349907	DB00763
**18**	54	Sphingosine	5280335	DB03203
**19**	55	Somatostatin	16129681	DB09099
**20**	56	Razoxane	30623	NA
**21**	57	Fludarabine	657237	DB01073
**22**	58	Reserpine	5770	DB00206
**23**	59	Benidipine	656668	DB09231
**24**	60	Ezetimibe	150311	DB00973
**25**	61	NBI-27914	176157	NA
**26**	62	KU-55933	5278396	NA
**27**	63	Azathioprine	2265	DB00993
**28**	64	Rescinnamine	5280954	DB01180

** 13 compounds highlighted in yellow are prioritized after our analysis.*

Table S6B. Grouping of 163 potential modulators of hyperinflammatory response into clusters based on their interaction patterns with their targets (*)

Cluster index	Index	Compound name	PubChem ID	DrugBank ID
**1**	1	Chlorprothixene	667467	DB01239
	2	Olanzapine	4585	DB00334
	3	Amoxapine	2170	DB00543
	4	Desipramine	2995	DB01151
	5	Trifluoperazine	5566	DB00831
	6	Maprotiline	4011	DB00934
	7	Promazine	4926	DB00420
	8	Ipsapirone	56971	NA
	9	Trazodone	5533	DB00656
	10	Nefazodone	4449	DB01149
	11	Zuclopenthixol	5311507	DB01624
	12	Cisapride	2769	DB00604
	13	Fluphenazine	3372	DB00623
	14	Lisuride	28864	DB00589
	15	Bromocriptine	31101	DB01200
	16	Piribedil	4850	DB12478
	17	Thioproperazine	9429	DB01622
	18	Quinpirole	54562	NA
	19	Pirenperone	4847	NA
	20	Ketanserin	3822	DB12465
	21	Latrepirdine	197033	DB11725
	22	Cyclazosin	132266	NA
	23	Midodrine	4195	DB00211
	24	Nor-binaltorphimine	5480230	NA
	25	EMD-386088	10131112	NA
	26	Dopamine	681	DB00988
	27	Azelastine	2267	DB00972
	28	Loratadine	3957	DB00455
	29	Verapamil	2520	DB00661
	30	Naftopidil	4418	DB12092
	31	Clonidine	2803	DB00575
	32	Alfuzosin	2092	DB00346
	33	Clebopride	2780	DB13511
	34	U-99194	5626	NA
**2**	35	Mepyramine	4992	DB06691
	36	Chlorphenamine	2725	DB01114
	37	Dicycloverine	3042	DB00804

	38	Oxybutynin	4634	DB01062
	39	Otenzepad	107867	NA
	40	Tolterodine	443879	DB01036
	41	Profenamine	3290	DB00392
	42	Xaliproden	NA	DB06393
	43	Alverine	3678	DB01616
	44	Bupropion	444	DB01156
	45	Indatraline	126280	NA
	46	Sertraline	68617	DB01104
**3**	47	Alprenolol	2119	DB00866
	48	ICI-89406	123686	NA
	49	Bisoprolol	2405	DB00612
	50	Carteolol	2583	DB00521
**4**	51	Clarithromycin	84029	DB01211
	52	Bepridil	2351	DB01244
**5**	53	Fipronil	3352	NA
	54	CGS-20625	163844	NA
	55	Topiramate	5284627	DB00273
	56	Chlordiazepoxide	2712	DB00475
**6**	57	Saracatinib	10302451	DB11805
	58	HY-11007	5311510	NA
	59	Dasatinib	3062316	DB01254
	60	Fostamatinib	11671467	DB12010
	61	TG-101348 (Fedratinib)	16722836	DB12500
	62	PLX-4720	24180719	DB06999
	63	TPCA-1	9903786	NA
	64	DMBI	5353593	NA
	65	Orantinib	5329099	DB12072
	66	D-64406	5330535	NA
	67	WZ-4002	44607530	NA
	68	bis-tyrphostin	5329255	NA
	69	JAK3-Inhibitor-II	3795	NA
**7**	70	TGX-221	9907093	NA
	71	AZD-6482	44137675	DB14980
	72	GDC-0941 (Pictilisib)	17755052	DB11663
	73	PI-103	9884685	NA
	74	AZD-8055	25262965	DB12774
**8**	75	Roscovitine (Seliciclib)	160355	DB06195
	76	CGP-60474	644215	NA
**9**	77	Milrinone	4197	DB00235
	78	Anagrelide	2182	DB00261
**10**	79	Hexamethylene	1794	NA
	80	FIT	84008	NA
	81	Loperamide	3955	DB00836
	82	BRL-52537	6603740	NA
**11**	83	Nicotine	89594	DB00184
	84	Zacopride	108182	NA
	85	Metoclopramide	4168	DB01233
	86	m-chlorophenylbiguanide	1354	NA
	87	Palonosetron	6337614	DB00377
	88	Alosetron	2099	DB00969
**12**	89	Flutamide	3397	DB00499
	90	Danazol	28417	DB01406
	91	Norgestimate	6540478	DB00957
	92	Oxybenzone	4632	DB01428
	93	α-estradiol	68570	NA
	94	Diethylstilbestrol	448537	DB00255

	95	Estradiol	5757	DB00783
	96	Testosterone	6013	DB00624
	97	Ticlopidine	5472	DB00208
	98	Nifedipine	4485	DB01115
	99	Nimodipine	4497	DB00393
	100	Spironolactone	5833	DB00421
	101	Liothyronine	5920	DB00279
	102	CITCO	9600409	NA
**13**	103	Piperine	638024	DB12582
	104	Phenelzine	3675	DB00780
	105	Iproniazid	3748	DB04818
	106	Selegiline	26757	DB01037
	107	Auraptene	1550607	NA
**14**	108	Atorvastatin	60823	DB01076
	109	Pravastatin	54687	DB00175
**15**	110	Exemestane	60198	DB00990
	111	Formestane	11273	DB08905
**16**	112	Felbamate	3331	DB00949
	113	Cycloserine	6234	DB00260
	114	Gavestinel	6450546	DB06741
	115	RO-25-6981	6604887	NA
**17**	116	EHNA	3206	NA
	117	SCH-442416	10668061	NA
**18**	118	Bromfenac	60726	DB00963
	119	DUP-697	3177	NA
	120	Dexketoprofen	667550	DB09214
	121	Oxaprozin	4614	DB00991
	122	Phenothiazine	7108	DB11447
	123	Triptolide	107985	DB12025
	124	Pyrazinamide	1046	DB00339
**19**	125	SCH-28080	108137	NA
	126	Pantoprazole	4679	DB00213
**20**	127	BIIB021	16736529	DB12359
	128	Geldanamycin	5288382	DB02424
**21**	129	STO-609	3467590	NA
**22**	130	Mafenide	3998	DB06795
**23**	131	AR-C133057XX	9797857	DB07002
**24**	132	Buphenine (Nylidrin)	4567	DB06152
**25**	133	AICA-ribonucleotide	65110	DB01700
**26**	134	Valproic acid	3121	DB00313
**27**	135	Iodophenpropit	3035746	NA
**28**	136	Retinol (Vitamin A)	445354	DB00162
**29**	137	Sildenafil	5212	DB00203
**30**	138	MK-2206	24964624	NA
**31**	139	KI-16425	10367662	NA
**32**	140	Dinoprostone	5280360	DB00917
**33**	141	BI-78D3	2747117	NA
**34**	142	Y-27632	448042	DB08756
**35**	143	SB-216763	176158	NA
**36**	144	Linsitinib	11640390	NA
**37**	145	PF-04217903	17754438	DB12848
**38**	146	Mepacrine	237	DB01103
**39**	147	Dicyclohexylurea	4277	NA
**40**	148	Pirfenidone	40632	DB04951
**41**	149	Dichloroacetic acid	6597	DB08809

**42**	150	MLN-4924 (Pevonedistat)	16720766	DB11759
**43**	151	Thenoyltrifluoroacetone	5601	DB04795
**44**	152	Navitoclax	24978538	DB12340
**45**	153	Daunorubicin	30323	DB00694
**46**	154	UNC-0321	46901937	NA
**47**	155	Isoliquiritigenin	638278	DB03285
**48**	156	PCA-4248	4698	NA
**49**	157	Pyroxamide	4996	DB12847
**50**	158	Rucaparib	9931954	DB12332
**51**	159	Ilomastat	132519	DB02255
**52**	160	Latrunculin-b	6436219	DB08080
**53**	161	Nicorandil	47528	DB09220
**54**	162	Z-prolyl-prolinal	122623	DB03535
**55**	163	BRD-K63784565	97226	DB12385

** The 24 compounds highlighted in yellow are prioritized based on the cluster analysis*

1 UniProt, C. UniProt: a worldwide hub of protein knowledge. *Nucleic Acids Res* **47**, D506-D515, doi:10.1093/nar/gky1049 (2019).
