# Supplementary material for: A systems‐level study reveals host‐targeted repurposable drugs against SARS‐CoV‐2 infection
Source: Mol Syst Biol. 2021 Aug 2;17(8):e10239. doi: 10.15252/msb.202110239 (PMC8328275; doi:10.15252/msb.202110239)
Supplement: Supplementary file 2 — Expanded View Figures PDF [file MSB-17-e10239-s003.pdf]

Expanded View Figures

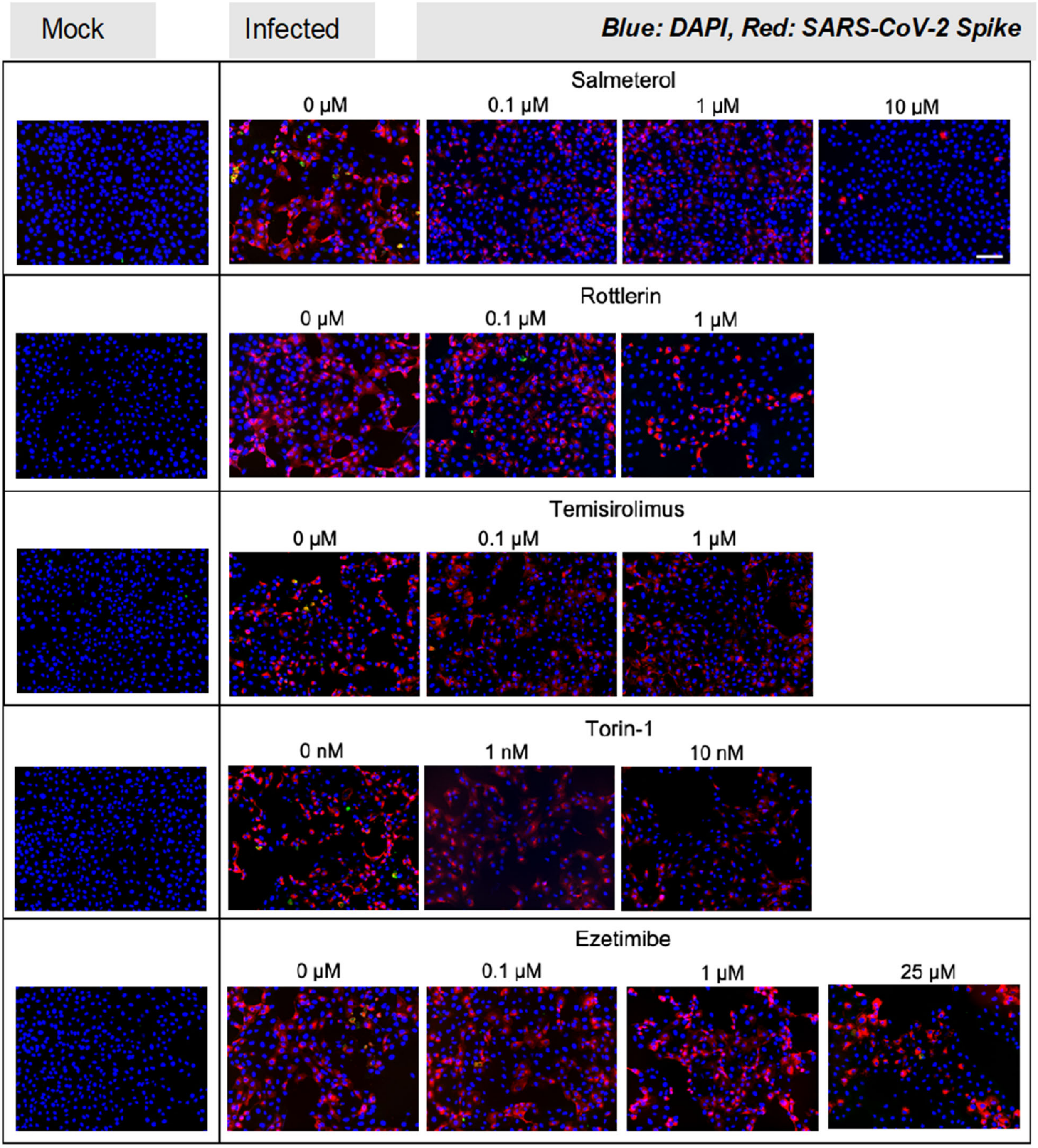

Figure EV1. Suppression of SARS-CoV-2 infection by identified compounds, related to Fig 5.

Vero-E6 cells were pretreated with compounds (salmeterol, rottlerin, temsirolimus, torin-1, or ezetimibe) for 1 h prior to SARS-CoV-2 inoculation. 48-h post-infection cells were fixed and labeled for SARS-CoV-2 S protein. Images are representative of five imaging fields in triplicate wells. Scale bar, 100 µm.

© 2021 The Authors
